# Efficient biodegradation and detoxification of reactive black 5 using a newly constructed bacterial consortium

**DOI:** 10.1186/s12934-025-02768-z

**Published:** 2025-07-02

**Authors:** Manar K. Abd Elnabi, Mohamed A. Ghazy, Sameh S. Ali, Marwa Eltarahony, Amr Nassrallah

**Affiliations:** 1 Biotechnology Program, Basic and Applied Science Institute, Egypt–Japan University of Science and Technology (E-JUST), New Borg El-Arab City, Alexandria 21934 Egypt; 2 Botany and Microbiology Department, Faculty of Science, Tanta University, Tanta, 31527 Egypt; 3 Biochemistry Department, Faculty of Science, Ain Shams University, Cairo, 11566 Egypt; 4 Environmental Biotechnology Department, Genetic Engineering and Biotechnology Research Institute (GEBRI), City of Scientific Research and Technological Applications (SRTA-City), New Borg El-Arab City, Alexandria 21934 Egypt; 5 Biochemistry Department, Faculty of Agriculture, Cairo University, Giza, 12613 Egypt

**Keywords:** Azo dye decolorization, Bacterial consortium, Bioremediation, Metabolite characterization, Statistical optimization, Toxicity reduction

## Abstract

The release of azo dyes into wastewater from textile industries poses a significant environmental challenge due to their toxicity and recalcitrance. Among these dyes, Reactive Black 5 (RB5) is one of the most widely used and environmentally persistent due to its complex aromatic structure, high stability, and extensive use in the textile sector. This study aimed to develop and optimize a highly efficient bacterial consortium for the decolorization and detoxification of RB5. Three bacterial species—*Bacillus cereus*, *Proteus mirabilis*, and *Stenotrophomonas maltophilia*—were isolated from industrial effluents and combined into a consortium based on compatibility testing. The optimization of cultural and incubation conditions using Plackett–Burman Design (PBD) and Central Composite Design (CCD) significantly enhanced RB5 decolorization efficiency, reaching 98.56% under static conditions. Enzymatic analysis revealed the crucial role of NADH-DCIP reductase and azoreductase in azo bond cleavage, while oxidative enzymes facilitated further degradation into non-toxic metabolites. Metabolite characterization using UV-Vis, FTIR, and GC-MS confirmed the breakdown of RB5 into intermediate compounds with reduced toxicity. Toxicity assessments demonstrated a 66.38–21.38% reduction in root growth inhibition, an increase in germination rate from 40 to 93.33%, a decrease in *Artemia salina* mortality from 86.7 to 23.3%, and a reduction in cytotoxicity from 55.31 to 14.45% in human breast epithelial cells. These findings demonstrate the potential of the developed consortium as an eco-friendly, cost-effective solution for RB5-contaminated wastewater. Future studies should focus on pilot-scale implementation, long-term stability under variable effluent conditions, and regulatory compliance for industrial deployment.

**Supplementary Information:**

The online version contains supplementary material available at 10.1186/s12934-025-02768-z.

## Introduction

The textile industry is a major contributor to environmental pollution due to the extensive use of synthetic dyes, especially azo dyes [1]. These compounds are favored for their vibrant colors, chemical stability, and cost-effectiveness. However, their widespread discharge into aquatic systems raises severe environmental and health concerns, as they are often toxic, mutagenic, and resistant to degradation [2]. Among them, Reactive Black 5 (RB5) is a widely used azo dye in the textile sector, known for its high tinctorial strength, solubility, and structural complexity [3]. Its resistance to biodegradation makes it a representative model for evaluating advanced bioremediation strategies [4]. Azo dyes account for over 70% of global synthetic dye production [5], and a large proportion enters wastewater streams due to incomplete fiber binding during dyeing processes [6]. These dyes persist in the environment, reduce light penetration in water bodies, disrupt aquatic photosynthesis, and affect both aquatic life and plant growth [7]. Some azo dye metabolites have also been linked to carcinogenicity [8].

Traditional treatment techniques—such as coagulation, membrane filtration, and advanced oxidation—are often costly, inefficient, and may produce secondary pollutants [9, 10]. In contrast, microbial bioremediation provides an eco-friendly and sustainable solution. Bacterial consortia, in particular, offer advantages over individual isolates through synergistic metabolic pathways and greater stability [4, 11]. Nevertheless, the high salinity commonly found in textile effluents can impair microbial activity, emphasizing the need for halotolerant or halophilic systems [12].

While many studies have explored RB5 biodegradation, few have employed an integrative approach that encompasses microbial isolation, enzymatic mechanism exploration, statistical process optimization, metabolite identification, and toxicity validation. Earlier research often relied on aerobic or stirred conditions, with decolorization efficiencies rarely exceeding 85% and limited attention given to the toxicity of degradation products [13]. By contrast, this study reports a high-efficiency (98.56%) decolorization system under static and saline conditions using a robust halotolerant bacterial consortium. The consortium was subjected to rigorous optimization using Plackett–Burman Design (PBD) and Central Composite Design (CCD), and its enzymatic activities were characterized to identify both reductive and oxidative degradation pathways. This study’s novelty lies in its comprehensive strategy that integrates the isolation of compatible halotolerant strains, application of advanced statistical modeling, enzymatic profiling, GC-MS-based metabolite analysis, and a multi-level toxicity evaluation including phytotoxicity, biotoxicity, and cytotoxicity.

To our knowledge, no previous study has combined all these aspects into a unified framework targeting RB5 degradation. This depth of analysis not only enhances mechanistic understanding but also improves the environmental relevance and industrial applicability of the proposed system. The ultimate objective was to establish a biologically effective, cost-efficient, and scalable bioremediation strategy for textile wastewater treatment. The outcomes of this study contribute directly to several United Nations Sustainable Development Goals (SDGs), including SDG 6 (Clean Water and Sanitation), SDG 12 (Responsible Consumption and Production), and SDG 13 (Climate Action), by promoting sustainable, non-toxic solutions to industrial pollution.

## Materials and methods

### Azo dye and microbiological medium

RB5, a sulfonic diazo dye with the molecular formula C₂₆H₂₁N₅Na₄O₁₉S₆, was obtained from Sigma Aldrich (USA). A mineral salt (MS) medium was used for the isolation of azo dye-degrading bacterial strains. The composition of the MS medium per liter of distilled water was as follows: 2 g NaCl, 1.5 g K₂HPO₄, 0.5 g KH₂PO₄, 0.2 g MgSO₄·7 H₂O, 0.05 g CaCl₂·2 H₂O, and 0.5 g (NH₄)₂SO₄. The pH of the medium was adjusted to 7. For solid media preparation, 2% agar was added. The composition of the MS medium was designed to support the growth of halotolerant bacteria while simulating the salinity of textile wastewater. Sodium chloride (NaCl) was included at 2 g/L as a baseline to support salt adaptation, and additional salinity stress experiments were conducted separately. To avoid interference with RB5 as the sole carbon source during initial screening, no external carbon or nitrogen sources were added [14]. However, in subsequent optimization studies, glucose was introduced as a co-substrate to assess its impact on decolorization efficiency [15]. The selection of this medium composition allowed for both the isolation of salt-tolerant, dye-degrading bacteria and the controlled assessment of metabolic enhancement under optimized conditions.

### Isolation of the RB5 Azo dye-decolorizing bacteria

Wastewater samples were collected from the final discharge point of a textile industry located in the 10th of Ramadan City industrial zone, Cairo, Egypt, before treatment at the effluent treatment plant. Samples were taken in sterile containers and transported immediately to the laboratory under refrigerated conditions (4 °C) for further processing. The enrichment method was used to screen and select bacterial strains capable of decolorizing RB5 [16]. Briefly, 10 mL of wastewater was inoculated into a 250 mL Erlenmeyer flask containing 90 mL of MS broth supplemented with 70 mg/L RB5 as the sole carbon source. An initial RB5 concentration of 70 mg/L was selected based on preliminary trials and literature reports as a suitable level that allows for visible decolorization without exerting excessive toxicity on microbial communities during enrichment [17]. This concentration effectively balanced selective pressure for azo dye degradation with survivability and growth of diverse bacterial strains. The culture was incubated at 35 °C for three days. The incubation temperature of 35 °C was selected based on prior studies reporting optimal bacterial growth and azo dye decolorization in this mesophilic range, as well as initial trials confirming effective RB5 decolorization at this temperature [18]. Following incubation, 10 mL of the cultured medium was transferred to a fresh MS broth medium with the same RB5 concentration and incubated for an additional three days. This enrichment process was repeated three times to establish a stable decolorization system. Three enrichment cycles were chosen based on the observation that decolorization performance plateaued after the third cycle, indicating stabilization of the microbial community. After each cycle, decolorization efficiency was monitored visually and spectrophotometrically, and no substantial increase in decolorization was observed beyond the third transfer. This suggested that the most active dye-degrading strains had become dominant, justifying the use of this point as the basis for isolation.

During enrichment and screening, RB5 was supplied as the sole carbon source in the MS medium to promote selective growth of dye-degrading bacteria. The successful isolation of decolorizing strains under the selective conditions strongly suggests their ability to metabolize the dye or its breakdown products [19]. Serial dilutions of the enriched cultures were prepared up to a 10⁻⁵ dilution. From each dilution, 100 µL was spread onto MS agar plates containing 70 mg/L RB5 and incubated at 35 °C until visible bacterial colonies formed. Control plates without RB5 were not included during isolation; however, the use of dye-containing MS agar as the sole carbon source acted as a strong selective pressure, favoring strains capable of tolerating and metabolizing the dye [20]. Only colonies showing visible decolorization zones on RB5-containing plates were selected for further study, ensuring functional relevance to dye degradation. These isolates were preserved in 20% glycerol and stored at − 80 °C for long-term use. As a result, three bacterial isolates were selected for subsequent experiments.

### Molecular characterization

The identification of the selected pure bacterial isolates was performed through 16S rRNA gene partial sequence analysis. Total DNA was extracted from the selected bacterial strains using the Quick-DNA™ Fungal/Bacterial Miniprep Kit (Zymo Research, USA). Polymerase chain reaction (PCR) amplification of the 16S rRNA gene was carried out using the COSMO Taq DNA Polymerase master mix (Willowfort, UK), following the manufacturer’s instructions. The universal primers used for PCR amplification were F- (5’- CAGGCCTAACACATGCAAGTC-3’) and R- (5’-CGGCGGWGTGTACAAGGC-3’). The PCR products were analyzed via 1% agarose gel electrophoresis, then purified [21]. The resulting DNA was sequenced using an ABI 3730 automated sequencer (PerkinElmer/Applied Biosystems, Foster City, CA, USA). The obtained sequences were compared with existing sequences in the NCBI GenBank database using the BLAST program. Additionally, a phylogenetic tree was constructed using MEGA7 software [22].

### Development of the bacterial consortium and compatibility assessment

The three bacterial species—*Bacillus cereus*, *Proteus mirabilis*, and *Stenotrophomonas maltophilia*—were selected based on their superior decolorization efficiency, observed through the formation of distinct clear zones on RB5-containing agar plates and rapid growth under dye-stressed conditions. Preliminary tests also showed that these isolates maintained stable decolorization activity across multiple enrichment cycles and under saline conditions, suggesting robust metabolic potential. Their combination into a consortium was guided by both their functional performance and compatibility, as confirmed by antagonism testing. To prepare the bacterial cultures, each strain was inoculated individually in nutrient broth and incubated in a shaking incubator at 35 °C with 120 rpm overnight. The optical density (OD_600_) of each culture was adjusted to 0.6. The bacterial consortium was constructed by combining the selected strains aseptically at equal cell densities [23]. To assess the compatibility between the selected strains, an antagonism test was performed to ensure that their interactions would not inhibit the decolorization process of RB5.

For the antagonism test, three nutrient agar plates were prepared. Two wells were made on each plate, and 100 µL of bacterial lawn (10⁸ CFU/mL) of each strain was inoculated separately using a sterile cotton swab. Each well was then inoculated with 100 µL of one of the other two bacterial strains. The plates were incubated at 35 °C for 24 h. Compatibility was confirmed by the absence of an inhibition zone around the wells, indicating no antagonistic interactions between the strains [24]. Following this, the developed bacterial consortium was used for subsequent experiments.

### Decolorization assay

The efficiency of the developed bacterial consortium in RB5 dye removal was evaluated by inoculating MS broth medium containing 70 mg/L RB5 with 5% (v/v) of the consortium. The cultures were incubated at 35 °C under both static and shaking (120 rpm) conditions for 48 h. An inoculum volume of 5% (v/v) was selected based on preliminary tests and literature precedent and showing that this amount ensured rapid bacterial adaptation and measurable decolorization within 48 h, without causing nutrient depletion or excessive biomass that could inhibit dye diffusion or enzyme activity [25, 26]. At 12-hour intervals, 5 mL of the culture was aseptically withdrawn from each flask and centrifuged at 5000 rpm for 15 min to separate the bacterial cell mass using a Sigma 3K15 refrigerated centrifuge (Sigma Laborzentrifugen GmbH, Germany). The residual dye concentration in the recovered supernatant was measured using a UV-Visible spectrophotometer (Model UV-1800, Shimadzu, Japan) at the maximum absorbance wavelength of RB5 (λmax = 597 nm). To ensure reproducibility and accuracy of UV-Vis measurements, all samples were centrifuged at 5000 rpm for 15 min prior to absorbance reading to remove suspended biomass and particulate matter that could interfere with spectrophotometric detection. Each absorbance measurement at RB5 (λmax = 597 nm) was performed in triplicate, and mean values were reported. Additionally, a control sample containing only the medium and RB5 (without inoculum) was used to account for any abiotic dye degradation or background absorbance, ensuring that observed changes were due solely to microbial activity [27, 28].

Preliminary trials revealed significantly higher decolorization under static conditions than under shaking. This effect is likely due to oxygen competition, where dissolved oxygen under shaking conditions inhibits the activity of key reductive enzymes, such as azoreductase and NADH-DCIP reductase, which require anaerobic or low-oxygen conditions for effective azo bond cleavage. Therefore, all subsequent experiments were conducted under static conditions. The decolorization efficiency of RB5 was calculated based on the reduction in absorbance of the supernatant at 597 nm [29], using the following equation:

$$\begin{aligned}\:& {\text{RB5}}\:{\text{decolorization}}\:\left( \% \right) \hfill \\ & \quad {\text{ = }}\left( {\frac{\begin{gathered}{\text{Initial}}\:{\text{absorbance}}\: \hfill \\{\text{ - }}\:{\text{Absorbance}}\:{\text{after}}\:{\text{decolorization}} \hfill \\ \end{gathered} }{{{\text{Initial}}\:{\text{absorbance}}}}} \right) \times \:{\text{100}} \hfill \\ \end{aligned} $$

Bacterial growth during the decolorization process was monitored using a spectrophotometer. After centrifugation, the bacterial cells were washed with a neutral phosphate buffer and centrifuged again at 5000 rpm for 10 min to separate the cell pellet. The pellet was then resuspended in sterile distilled water, and the OD_600_ nm was measured to assess bacterial growth.

### Optimization of RB5 azo dye decoloriztion conditions

The optimization of cultural conditions for enhancing the decolorization performance of the bacterial consortium was performed using a statistical design of experiments (DOE) approach, which aimed to improve dye decolorization efficiency while minimizing process costs. The DOE was conducted in two stages: first, using the PBD to screen for significant factors, and then applying the CCD for further optimization. All experimental trials for RB5 decolorization by the developed consortium were carried out in 100 mL Erlenmeyer flasks containing 50 mL of MS broth medium under static conditions.

#### Plackett-burman design

The PBD is an efficient method for screening a large number of input factors with a reduced number of experimental runs, compared to traditional one-factor-at-a-time approaches [30]. In this study, significant variables affecting the decolorization of RB5 were selected based on their main effects. Each independent factor was evaluated at both high (+) and low (−) levels, as shown in Table 1. The PBD matrix was developed for twelve independent factors, which were tested through 20 experimental runs (Table S1). Each column of the matrix contains an equal number of (+) and (−) signs. The main effect of each parameter on RB5 decolorization was calculated as the difference in response values between the high and low levels. PBD is depended on the first order polynomial law as represented in the following equation [31]:

**Table 1:** Factor levels tested in the screening design

Factors	Experimental value	
	Low (-1)	High (+ 1)
Dye concentration (mg/L)	50	120
Glucose (g/L)	2	10
(NH_4_)_2_SO_4_ (g/L)	0.25	1
MgSO_4_ (g/L)	0.1	0.3
NaCl (g/L)	1	3
CaCl_2_ (g/L)	0.025	0.1
K_2_HPO_4_ (g/L)	0.75	3
KH_2_PO_4_ (g/L)	0.25	1
pH	5	8
Temperature (^°^C)	30	40
Inoculum size (%)	2	7
Incubation time (hour)	24	72

$${\rm{Y = \beta 0}}\>{\rm{ + }}\sum {{\rm{\beta iXi}}} $$

Where Y is the RB5 decolorization %, β0 and βi are the model intercept and linear coefficient, respectively, and Xi is the level of each independent variable.

The decolorization rate of RB5 was measured for each experimental trial. Variables with a significant impact on RB5 decolorization (*P* < 0.05) as determined by regression analysis were identified. These significant factors were then further optimized using the CCD.

#### Response surface methodology using central composite design

The effects of four significant factors identified from the PBD—RB5 concentration, pH, inoculum size, and incubation time—on RB5 decolorization were further analyzed using the CCD. The CCD matrix consisted of 31 experimental runs (Table S2), with each independent factor tested at five levels: −2, − 1, 0, 1, and 2 (Table 2). The biodegradation of RB5 was investigated by the second order polynomial law as represented in the following equation [32]:

**Table 2:** Variables tested in the CCD

Variable	Coded levels/experimental values				
	-2	-1	0	+ 1	+ 2
RB5 concentration (mg/L)	10	30	50	80	100
pH	5	7	8	9	10
Inoculum size (%)	2	5	7	10	12
Incubation time (h)	24	48	72	96	120

$$\eqalign{& Y = \beta 0 + \beta 1X1 + \beta 2X2 + \beta 3X3 + \beta 11{(X1)^2} \cr & + \beta 22{(X2)^2} + \beta 33{(X3)^2} + \beta 12X1X2 + \beta 13X1X3 + \beta 23X2X3 \cr} $$

Where Y is the predicted response (decolorization percentage); X1, X2, X3 are the coded values of significant independent variables which influence the response Y; β0 is the constant coefficient; β1, β2 and β3 are the linear coefficients; β11, β22 and β33 represent the squared or quadratic regression coefficients; β12, β13, and β23 denote the interaction coefficients.

#### Validation of the statistical model

The developed statistical model was validated through a confirmation experiment conducted in the laboratory. This experiment aimed to compare the actual RB5 biodegradation under the predicted optimal conditions with the maximum RB5 removal predicted by the statistical design. Additionally, the decolorization efficiency of RB5 under optimized conditions was compared with that under basal (pre-optimization) conditions to assess the accuracy and effectiveness of the model.

### Decolorization of RB5 under salinity stress

The effect of hypersaline conditions on RB5 decolorization by the developed bacterial consortium was evaluated by varying the salt concentration in the range of 0–50 g/L while maintaining all other conditions at their optimal levels. Aliquots were collected at regular time intervals over a 120-hour incubation period and analyzed for both decolorization percentage and bacterial consortium growth rate.

### Enzyme activity assays

Under optimized conditions, the activity of key oxidoreductase enzymes involved in RB5 biodegradation by the bacterial consortium was assessed spectrophotometrically. The enzymes studied included NADH-DCIP reductase, azoreductase, lignin peroxidase (LiP), manganese peroxidase (MnP), and laccase (Lac). After the decolorization process, bacterial cells were collected by centrifugation and used as a source of intracellular enzymes. The cell pellets were resuspended in potassium phosphate buffer (50 mM, pH 7.4) and sonicated at 4 °C. The sonicated cell suspension was then centrifuged at 5000 rpm for 15 min at 4 °C, and the resulting supernatant was used as the crude enzyme extract for all enzymatic assays. The activities of LiP, MnP, and Lac were determined using veratryl alcohol, manganese sulfate, and 2,2-azonodi-3-ethylbenzothiazoline-6-sulfonic acid (ABTS) as substrates, respectively [33, 34]. NADH-DCIP reductase activity was measured by monitoring the reduction of dichlorophenol indophenol (DCIP) at 590 nm, while azoreductase activity was determined by monitoring the reduction of methyl red at 430 nm [35]. One unit of enzyme activity was defined as the amount of enzyme required to catalyze the conversion of 1 µM of substrate per minute per milligram of protein.

### Analysis of metabolites produced from RB5 azo dye biodegradation

The metabolic products resulting from the biodegradation of RB5 under optimal conditions were analyzed and compared with the undegraded dye. Upon complete degradation, 100 mL of the decolorized culture medium was centrifuged at 5000 rpm for 15 min at 4 °C. The metabolites in the supernatant were extracted three times with an equal volume of ethyl acetate, dried over anhydrous sodium sulfate, and concentrated using a rotary evaporator at 40 °C [36]. The final solution was filtered through a 0.22 μm syringe filter and prepared for analysis.

The variation in the absorption spectra of RB5 before and after biodegradation was analyzed using a Perkin Elmer Lambda 4B UV/Vis spectrophotometer in the range of 250 to 800 nm. Fourier Transform Infrared (FTIR) spectroscopy was performed to identify the characteristic functional groups in the untreated dye and its biodegraded products, using a Perkin-Elmer 1430 FT/IR spectrophotometer (PerkinElmer, USA). The FTIR measurements were conducted in the infrared spectral region of 400–4000 cm⁻¹.

For further metabolite identification, Gas Chromatography-Mass Spectrometry (GC-MS; Shimadzu, Japan) was used to analyze the degraded metabolites formed during RB5 biodegradation. A Claurus 580/560S GC-MS system was employed, with separation achieved using an Rtx-5MS column (0.25 μm thickness, 30 m length, 0.25 mm diameter). The GC operational conditions were applied as described previously [37]. The intermediates of RB5 degradation were identified by comparing their mass spectra with the standard spectra in the National Institute of Standards and Technology (NIST) library database.

### Toxicity studies

#### Phytotoxicity

The phytotoxic effects of the untreated RB5 dye (85 mg/L) and its biodegradation metabolites were evaluated on the growth of *Triticum aestivum*, an economically important agricultural plant. Briefly, uniform, healthy seeds of the plant were placed on a sterilized 5.5 cm filter paper in a petri dish, which was then filled with 3 mL of the test solution—either untreated RB5 or its biodegraded metabolites. The petri dishes were covered to maintain moisture and incubated in complete darkness for 3 days at room temperature. Distilled water was used as a control. At the end of the incubation period, the root length was measured, and the root growth inhibition was calculated based on the comparison between treated and control samples. The plumule length was measured and the percent of germination was calculated as the ratio between the numbers of germinated seeds to total number of the seeds [38].

$$\begin{aligned}&\text{Inhibition}\:\left(\text{\%}\right)\\ & =\left(\frac{\text{Root length of control - Root length of dye solution}}{\text{Root length of control}}\right)\text{}\times100 \end{aligned}$$

#### Biotoxicity assay

The toxicity of the untreated RB5 dye (85 mg/L) and its biodegradation metabolites was tested using newly hatched (24-hour-old) nauplii of *Artemia salina*. Test tubes were prepared, each containing 2 mL of artificial seawater and 3 mL of either untreated RB5 dye or its biodegradation metabolites. Ten nauplii were added to each test tube, and the tubes were incubated at 28 °C for 2 days. The control group consisted of artificial seawater with *Artemia*, without any dye. After the incubation period, the number of dead nauplii was counted, and the mortality percentage was calculated based on the comparison between treated and control groups [39].

$$\:\text{Mortality}\left(\text{\%}\right)\:\text{=}\:\left(\text{}\frac{\text{Number of dead}\:\text{Artemia}\:}{\text{Initial number of live}\:\text{Artemia}\:}\text{}\right)\times100$$

#### Cytotoxicity with human cell lines

The cytotoxicity of untreated RB5 dye and its biodegradation metabolites was assessed against two normal human cell lines: normal skin fibroblast BJ-1 (ATCC CRL-2522™) and epithelial breast cell MCF-12 F (ATCC CRL-10782™). Both cell lines were maintained in DMEM medium containing 2 mM L-glutamine (Biowest, USA). For the cytotoxicity test, cell viability was evaluated using the MTT assay [40]. Cells were seeded in 96-well microtiter plates at a density of 1 × 10⁴ cells per well and incubated for 24 h at 37 °C in a 5% CO₂ atmosphere with 95% humidity. After incubation, the cells were exposed to different concentrations (0–50%) of untreated RB5 (85 mg/L) and its biodegradation metabolites, and incubated for 48 h. DMEM alone served as the negative control. Following exposure, 40 µL of MTT solution (Bio Basic Canada Inc, Canada) at 2.5 µg/mL was added to each well, and the plates were incubated for an additional 4 h at 37 °C in a 5% CO₂ incubator. Then, 200 µL of 10% sodium dodecyl sulfate was added to each well, and the plates were incubated overnight at 37 °C to stop the reaction and dissolve the formazan crystals. The colored formazan product (purple) was quantified using a microplate reader (Bio-Rad Model 3550, USA) at 595 nm. The percentage of cytotoxicity was determined based on the absorbance values of treated cells relative to the control. The IC_50_ (concentration causing 50% death of the cells) was also calculated [40].

### Statistical analysis

All experiments were performed in triplicate, and the collected data were statistically analyzed using one-way analysis of variance (ANOVA) to compare the effects of different treatments. A significance level of *P* ≤ 0.05 was considered statistically significant. For optimization studies, data were analyzed using Minitab 14.0 (Minitab Inc., Pennsylvania, USA) to ensure a comprehensive and straightforward interpretation of results.

## Results and discussion

### Isolation and identification of the most potent RB5 azo dye-decolorizing bacteria

**Fig. 1:**
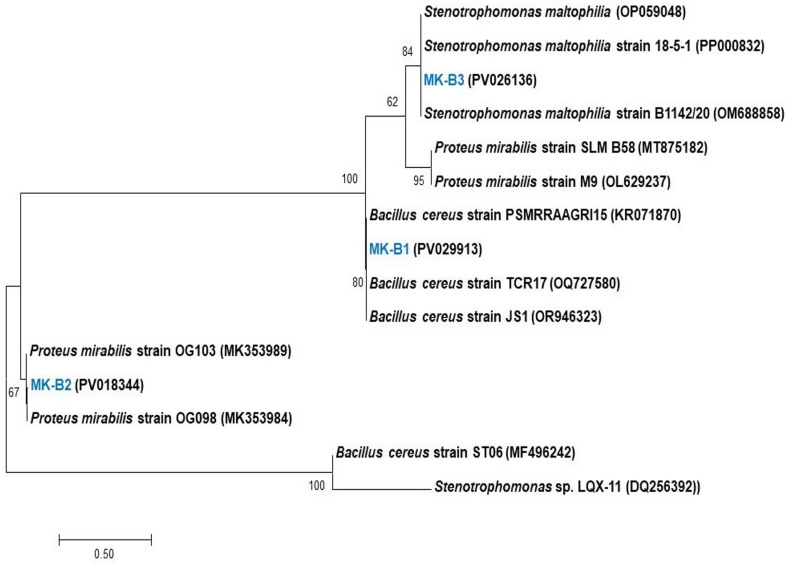
Neighbor-joining phylogenetic tree of the three representive bacterial strains of developed consortium and their related species based on genebank database

After preliminary screening, effluent samples collected from textile industry discharge sites were enriched, resulting in the isolation of 32 bacterial strains capable of degrading the azo dye RB5. Among these, three isolates exhibited the fastest growth rates and the largest decolorization zones, suggesting their superior decolorization potential. Consequently, the individual decolorization efficiency of these isolates against RB5 (70 mg/L) was quantitatively assessed, yielding efficiencies of 38.55%, 45.13%, and 43.38% for isolates MK-B1, MK-B2, and MK-B3, respectively. The selected isolates were subsequently identified via 16 S rRNA gene sequencing as *Bacillus cereus* (MK-B1), *Proteus mirabilis* (MK-B2), and *Stenotrophomonas maltophilia* (MK-B3). Their corresponding gene sequences were deposited in GenBank under accession numbers PV029913, PV018344, and PV026136, respectively. To further elucidate their phylogenetic relationships, a neighbor-joining phylogenetic tree was constructed (Fig. 1). Notably, the capacity of these bacterial species for azo dye bioremediation has been previously documented. For example, *Bacillus cereus* HJ-1 was reported to effectively decolorize RB5 [41]. Likewise, *Proteus mirabilis* LAG demonstrated the ability to degrade Reactive Blue 13 azo dye [42]. Additionally, Vilchis-Carmona et al. [43] reported that two strains of *Stenotrophomonas* (TepeL and TepeS) exhibited robust decolorization activity against five different textile azo dyes.

### Consortium synergism assessment and its performance on RB5 decolorization

**Fig. 2:**
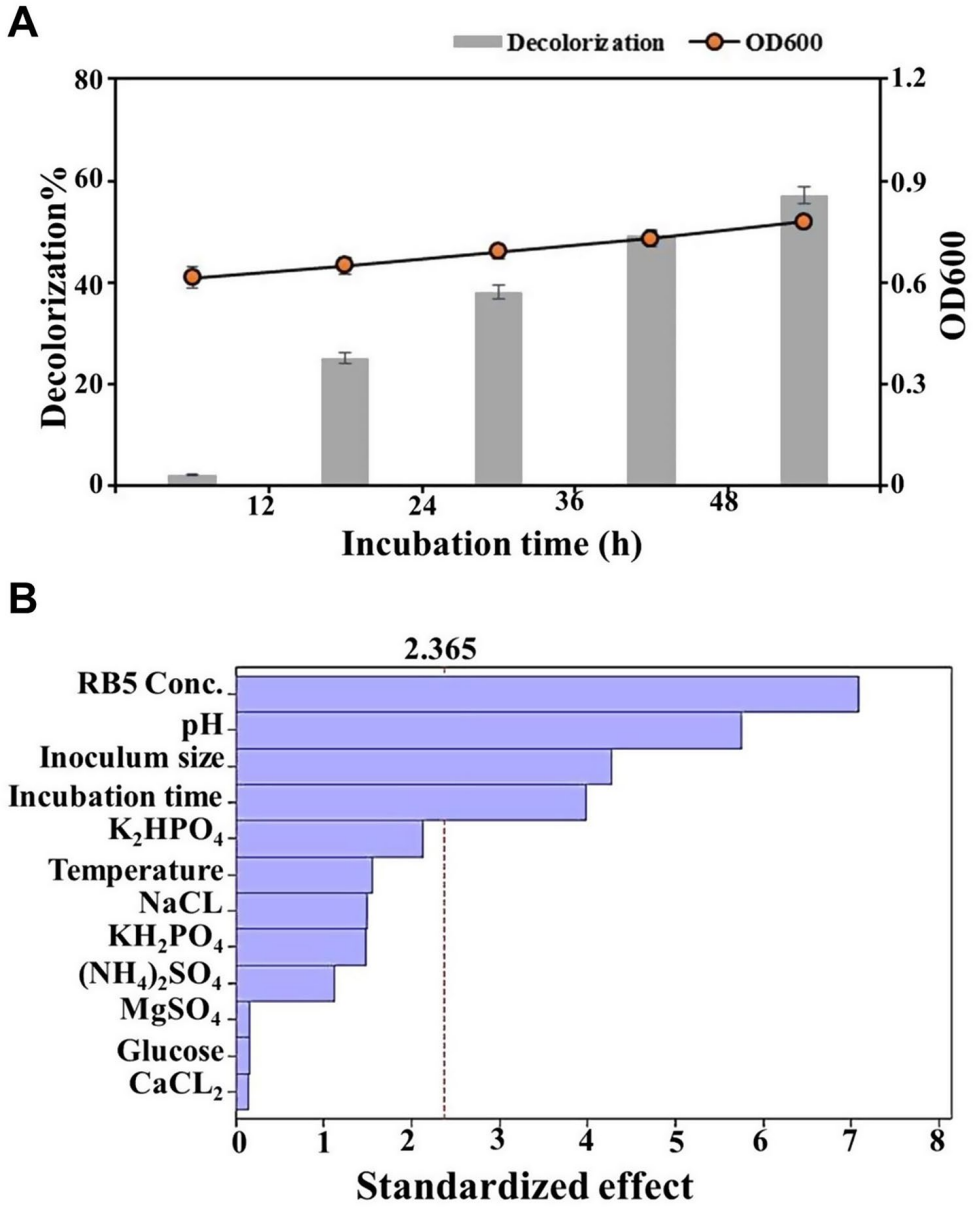
Assessment of RB5 decolorization performance and identification of key influencing factors. **(A)** Decolorization percentage and bacterial growth (OD600) measured at different incubation times, showing a time-dependent increase in both parameters. **(B)** Pareto chart of standardized effects identifying the most significant variables affecting RB5 decolorization, with dye concentration, pH, and inoculum size being the most influential

A compatibility test was conducted to assess whether the selected bacterial strains could function efficiently as a consortium for RB5 biodegradation. No inhibition zones were observed around the wells on any of the cultivated plates after incubation, indicating the absence of antagonistic interactions among the strains. This compatibility is likely due to the natural adaptation of these strains, which have co-existed in textile wastewater environments for an extended period. Their synergistic relationship suggests that they would be highly effective in RB5 azo dye degradation. Following confirmation of compatibility, the bacterial consortium was developed and tested for RB5 removal. The results demonstrated that decolorization efficiency was significantly higher under static conditions than under agitation. Specifically, RB5 biodegradation (70 mg/L) reached 57.21% under static conditions (Fig. 2A). In contrast, decolorization dropped sharply to less than 10% under shaking conditions (data not shown). The superior performance of static conditions in RB5 decolorization can be attributed to the metabolic characteristics of the bacterial consortium and the enzymatic pathways involved [44]. Under static (low-oxygen) conditions, the expression and activity of reductive enzymes, such as azoreductase and NADH-DCIP reductase, are typically enhanced. These enzymes are responsible for the initial cleavage of azo bonds, which requires reducing equivalents (e.g., NADH or NADPH) rather than molecular oxygen [45]. In contrast, shaking conditions introduce higher levels of dissolved oxygen, which not only suppresses the expression of these reductive enzymes but also promotes aerobic respiration over reductive dye metabolism [18]. It has been reported that only 13% of RB5 was decolorized by *Sterigmatomyces halophilus* under shaking conditions [28]. Similarly, *Bacillus pseudomycoides* could only remove 11.23% of Acid Black 24 in an agitated culture [46]. These studies support the observation that static conditions favor azo dye degradation, reinforcing the necessity of optimizing the bioremediation process for enhanced efficiency. Additionally, under aerobic conditions, oxygen may act as a competing electron acceptor, reducing the availability of electrons for azo bond reduction [47]. This metabolic shift diminishes the efficiency of dye degradation. The observed increase in enzyme activity under static conditions further supports this interpretation, highlighting the consortium’s preference for facultative anaerobic metabolism during RB5 decolorization. As a result, all subsequent experiments were conducted under static conditions.

### Optimization of RB5 decolorization conditions

#### Screening of significant variables influencing the RB5 removal

In this study, PBD was employed to identify the most influential parameters affecting the optimization of the RB5 decolorization process. As presented in Table S1, the response values for RB5 removal varied significantly, ranging from 6.78% (trial number 2) to 90.0% (trial number 15). This substantial variation underscores the necessity of optimizing process conditions to enhance decolorization efficiency. The impact of 12 independent variables on RB5 decolorization was statistically analyzed using PBD. The coefficients of each variable, which indicate their effect on RB5 removal, along with the corresponding p-values, are summarized in Table 3. Based on these statistical results, four parameters were identified as significantly influencing RB5 decolorization: RB5 concentration (P-value = 0.000), inoculum size (P-value = 0.004), pH (P-value = 0.001), and incubation time (P-value = 0.005). These parameters were subsequently selected for further optimization using CCD.

**Table 3:** Statistical analysis of parameters affecting decolorization of RB5 using PBD

Term	Coef	SE Coef	T	*P*
Constant	56.23	2.381	23.62	0.000
RB5 concentration	-16.87	2.381	− 7.09	0.000
Glucose	− 0.335	2.381	− 0.15	0.886
(NH_4_)_2_SO_4_	2.657	2.381	1.12	0.301
NaCl	3.544	2.381	1.49	0.180
MgSO_4_	0.377	2.381	0.16	0.879
CaCl_2_	-0.338	2.381	− 0.14	0.891
K_2_HPO_4_	5.044	2.381	2.12	0.072
KH_2_PO_4_	− 3.527	2.381	-1.48	0.182
pH	13.69	2.381	5.75	0.001
Temperature	3.683	2.381	1.55	0.166
Inoculum size	10.16	2.381	4.27	0.004
Incubation time	9.478	2.381	3.98	0.005

**Coef**, estimated coefficient; **SE Coef**, standard errors of the coefficient; **T**, t-statistics; **P**, level of significance

Conversely, factors such as glucose, (NH₄)₂SO₄, MgSO₄, NaCl, CaCl₂, K₂HPO₄, KH₂PO₄, and temperature were found to be statistically insignificant (*P* > 0.05) for RB5 removal. This finding was further validated by the Pareto chart (Fig. 2B), which ranked the relative significance of each parameter. Additionally, Fig. S1 illustrates the main effects of each factor on RB5 decolorization by the bacterial consortium. The statistical analysis of the PBD model, as presented in Table 4, confirmed its high significance, with a P-value of 0.002. The robustness and predictive power of the model were further demonstrated by a coefficient of determination (R²) of 94.9%, indicating a strong correlation between observed and predicted data and confirming the model’s reliability [48]. Based on PBD analysis, a first-order regression model was derived to describe the RB5 decolorization process using the results of 20 experimental trials. The model equation is expressed as follows: RB5 removal (%) = 56.23–16.87 (RB5 concentration) − 0.355 (Glucose) + 2.657 (NH_4_)2SO_4_ + 3.544 (NaCl) + 0.377 (MgSO_4_) − 0.338 (CaCl_2_) + 5.044 (K_2_HPO_4_) – 3.527 (KH_2_PO_4_) + 13.69 (pH) + 3.683 (Temperature) + 10.16 (Inoculum size) + 9.478 (Incubation time). This model provides insight into the role of each parameter in RB5 decolorization and serves as a foundation for further optimization using CCD.

**Table 4:** Analysis of variance for RB5 decolorization by tested consortium using PBD

Source	DF	Seq SS	Adj SS	Adj MS	F	*P*
Main Effects	12	14735.2	14735.2	1227.9	10.83	0.002
Residual Error	7	793.6	793.6	113.4		
Total	19	15528.7				

**DF**, Degrees of freedom; **Seq SS**, Sequential sum of square; **Adj SS**, Adjusted sum of squares; **Adj MS**, Adjusted mean square; **F**, Fishers’s function; **P**, probability of significance

Previous studies have emphasized the significance of pH, dye concentration, inoculum size, and incubation time in the optimization of azo dye decolorization [49, 50]. These critical parameters were further investigated to determine their optimal levels. A crucial aspect of azo dye biodegradation is the availability of a suitable carbon source. Due to their low carbon content, azo dyes often require an additional carbon source to enhance microbial degradation [51]. In this study, glucose was introduced as a co-substrate to improve RB5 decolorization efficiency.

Interestingly, results indicated that lower glucose concentrations enhanced decolorization efficiency, whereas higher glucose concentrations inhibited the process. This inhibitory effect may be attributed to the rapid accumulation of organic acids in the decolorization medium, leading to pH reduction and subsequent inhibition of bacterial growth and enzyme activity [26]. Additionally, glucose supplementation did not significantly affect RB5 decolorization efficiency in *Bacillus cereus* [17]. Another important factor influencing the decolorization process is temperature. Since bacterial growth and enzymatic activity are temperature-dependent, variations in temperature can significantly affect biodegradation efficiency [52]. Previous research suggests that the optimal temperature range for azo dye decolorization is between 30 and 40 °C [18]. In this study, the highest decolorization efficiency was observed at 40 °C, with both lower and higher temperatures reducing efficiency. This decline can be attributed to a reduction in microbial activity at lower temperatures and enzyme inactivation at excessively high temperatures [53]. These findings emphasize the importance of maintaining optimal temperature conditions for maximizing RB5 removal.

For the subsequent CCD optimization stage, factors that exhibited a positive effect (+) on RB5 decolorization were set at their high levels, while those that had a negative effect (-) were maintained at their low levels. This strategy ensures that the most influential parameters are fine-tuned to maximize RB5 decolorization efficiency. Overall, the PBD screening successfully identified four key factors—RB5 concentration, inoculum size, pH, and incubation time—that significantly influenced RB5 decolorization. The statistical model demonstrated high predictive accuracy (R² = 94.9%), confirming its reliability in optimizing decolorization conditions. Additionally, results highlighted the impact of glucose concentration on RB5 removal, with lower levels proving more effective due to pH stabilization. Temperature optimization was also identified as a critical parameter, with the highest efficiency observed at 40 °C. The next phase of the study will involve CCD optimization, where the identified significant parameters will be further refined to achieve the maximum biodegradation efficiency of RB5.

#### Central composite design for optimization of RB5 removal

CCD was employed to optimize the significant factors identified via PBD and to explore the interactive effects of these variables on the biodegradation of RB5. A 31-trial CCD matrix was used to test four key factors—RB5 concentration, pH, inoculum size, and incubation time—at five different levels. The CCD matrix, which includes the coded and actual levels of each selected variable, experimental and predicted responses for RB5 removal, and standardized residuals (Table S2). Decolorization of RB5 varied considerably among the experimental trials, with the highest response (93.84% RB5 decolorization) observed in trial 10 and the lowest (28.56%) in trial 25, indicating the interactive effects of the parameters across various levels. The decolorization rates were statistically analyzed using multiple regression analysis and ANOVA (Tables 5 and 6). The decolorization kinetics were indirectly assessed across a range of initial RB5 concentrations during CCD-based optimization, where dye concentration was tested at five levels (60 to 110 mg/L). The response surface plots revealed that decolorization efficiency decreased at higher RB5 concentrations, likely due to saturation of active enzyme sites and increased toxicity to bacterial cells. The maximum decolorization efficiency (93.84%) was observed at 85 mg/L RB5, which was determined to be the optimal concentration. Beyond this level, degradation declined markedly, consistent with the inhibitory effects reported for high dye loads in azo dye systems [54]. These results confirm that RB5 concentration is a critical parameter influencing biodegradation kinetics.

**Table 5:** Estimated regression coefficients, T–test and P–values of variables for the optimization of RB5 removal using CCD

Term	Coef	SE Coef	T	*P*
Constant	74.25	1.526	48.63	0.000
RB5	-8.384	0.824	-10.16	0.000
pH	6.029	0.824	7.313	0.000
Inoculum size	6.777	0.824	8.220	0.000
Incubation time	7.894	0.824	9.575	0.000
(RB5)^2^	-2.174	0.755	-2.879	0.011
(pH)^2^	-2.918	0.755	-3.864	0.001
(Inoculum size)^2^	-1.787	0.755	-2.366	0.031
(Incubation time)^2^	-0.226	0.755	-0.299	0.769
RB5 × pH	1.850	1.009	1.833	0.086
RB5 × Inoculum size	0.643	1.009	0.637	0.533
RB5 × Incubation time	2.758	1.009	2.731	0.015
pH × Inoculum size	-0.134	1.009	− 0.133	0.896
pH × Incubation time	0.648	1.009	0.642	0.530
Inoculum size × Incubation time	1.053	1.009	1.043	0.312

**Coef**, estimated coefficient; **SE Coef**, standard errors of the coefficient; **T**, t-statistics; **P**, level of significance

**Table 6:** Analysis of variance for RB5 removal using CCD

Source	DF	Seq SS	Adj SS	Adj MS	F	*P*
Regression	14	5763.19	5763.19	411.66	25.23	0.000
Linear	4	5157.85	5157.85	1289.46	79.03	0.000
Square	4	397.46	397.46	99.36	6.09	0.004
Interaction	6	207.89	207.89	34.65	2.12	0.107
Residual error	16	261.04	261.04	16.32		
Lack of fit	10	215.84	215.84	21.58	2.87	0.105
Pure error	6	45.20	45.20	7.53		
Total	30	6024.24				

**DF**, Degrees of freedom; **Seq SS**, Sequential sum of square; **Adj SS**, Adjusted sum of squares; **Adj MS**, Adjusted mean square; **F**, Fishers’s function; **P**, probability of significance

The regression analysis and model evaluation were performed using R² and adjusted R² (R² adj) values, along with Fisher’s test (F-value), lack-of-fit, and p-value. The regression model was statistically significant, as confirmed by an F-value of 25.23 and a corresponding P-value < 0.001, indicating that the variation in RB5 decolorization was strongly influenced by the selected factors. The high coefficient of determination (R² = 95.7%) and adjusted R² (92%) demonstrated excellent agreement between experimental and predicted values, supporting the model’s reliability. Additionally, the lack-of-fit test was non-significant (*P* = 0.105), further confirming the model’s adequacy and suggesting no substantial unexplained variation. These results validate the robustness of the CCD-based model for predicting and optimizing RB5 decolorization.

**Fig. 3:**
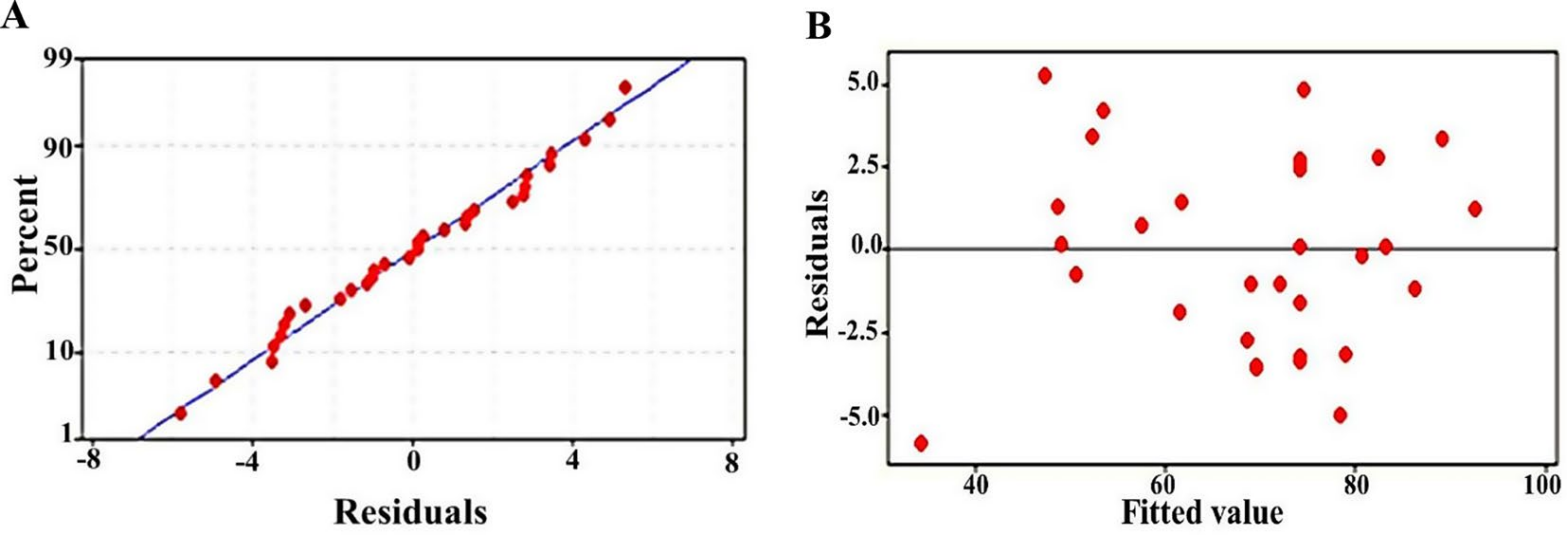
Diagnostic plots evaluating the adequacy of the statistical model used for RB5 decolorization analysis. **(A)** Normal probability plot of residuals indicating that the residuals follow a normal distribution, as the points align closely along the straight line. **(B)** Plot of residuals versus fitted values showing random scatter, suggesting homoscedasticity and confirming that the model fits the data without systematic error

The normal probability plot (Fig. 3A) also confirmed the suitability of the model, where the data points aligned closely with the straight line, indicating normally distributed residuals. Additionally, the residuals plotted against the fitted values (Fig. 3B) showed a random distribution around the center line, further confirming the model’s appropriateness. The multiple regression analysis revealed both linear and quadratic effects of the factors, as well as their interactions on RB5 decolorization. The linear effects of each variable were significant (*P* < 0.00), and quadratic effects were significant for most variables, except for incubation time (*P* = 0.769). Interaction effects among variables were mostly non-significant (*P* > 0.05), except for the interaction between RB5 concentration and incubation time (*P* = 0.015) as shown in Table 5. Synergistic effects were observed in the following variable pairs: RB5 concentration and pH, RB5 concentration and incubation time, RB5 concentration and inoculum size, incubation time and pH, and incubation time and inoculum size. Positive coefficients for these interactions indicated that increasing the concentration of both interacting variables resulted in a higher decolorization response. In contrast, the interaction between pH and inoculum size was antagonistic, as a higher RB5 decolorization was achieved by reducing one variable while increasing the other. The optimized model for RB5 removal is represented by the following second-order polynomial equation: RB5 degradation (%) = 74.25–8.384 RB5 + 6.029 pH + 6.777 Inoculum size + 7.894 Incubation time − 2.174 (RB5)^2^ − 2.918 (pH)^2^ − 1.787 (Inoculum size)^2^ − 0.226 (Incubation time)^2^ +1.850 RB5 × pH + 0.643 RB5 × Inoculum size + 2.758 RB5 × Incubation time − 0.134 pH ×Inoculum size + 0.648 pH × Incubation time + 1.053 Inoculum size × Incubation time.

**Fig. 4:**
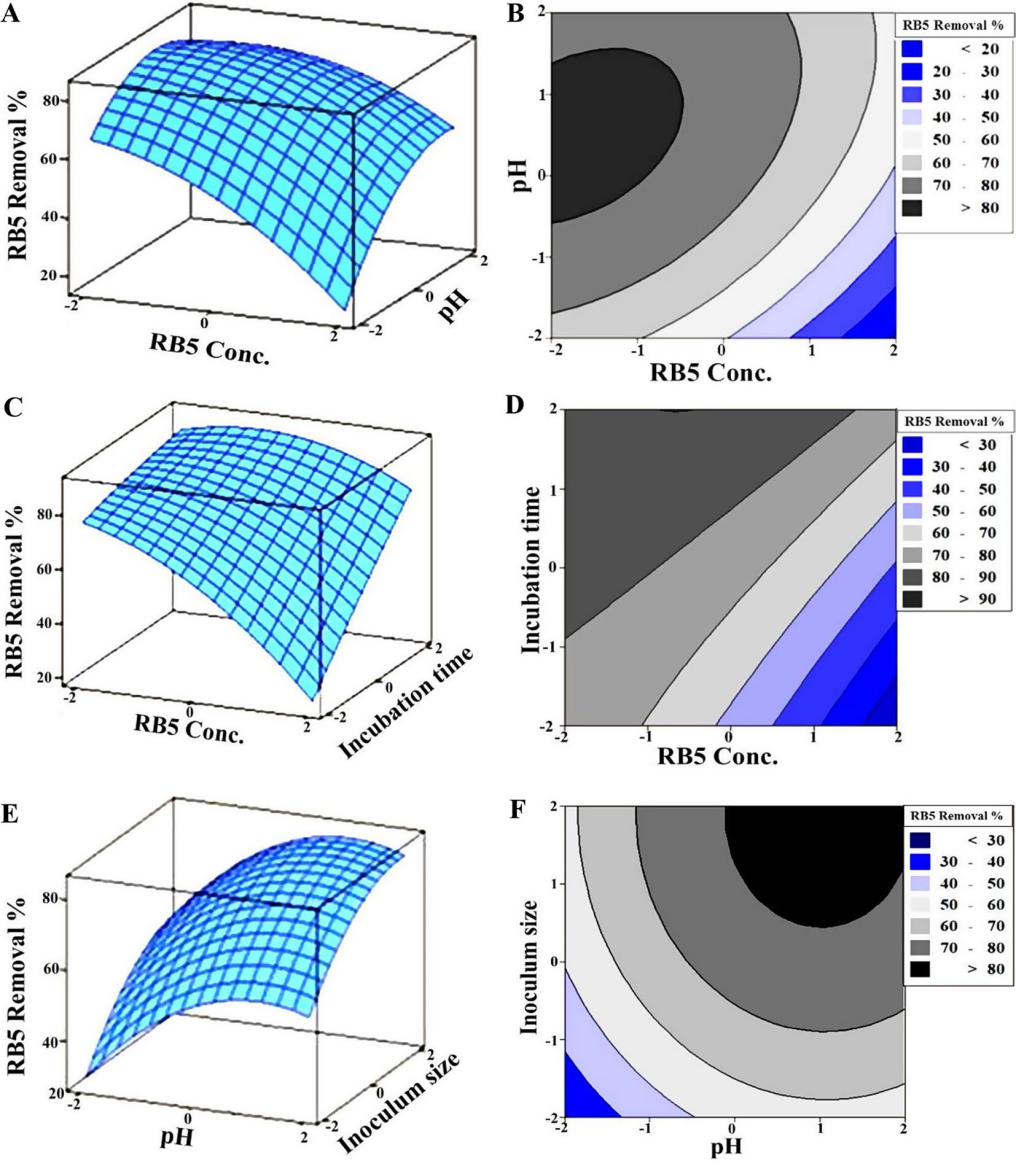
Three-dimensional surface plots and corresponding contour plots showing the interactive effects of key variables on RB5 decolorization efficiency. **(A**,** B)** Effect of RB5 concentration and pH on RB5 removal (%), indicating a decrease in removal efficiency at higher dye concentrations and suboptimal pH levels. **(C**,** D)** Effect of RB5 concentration and incubation time, showing improved decolorization with longer incubation, especially at lower dye concentrations. **(E**,** F)** Effect of pH and inoculum size, demonstrating enhanced dye removal under mildly acidic conditions and higher inoculum levels

To better understand the effect of interactions, the optimal values and the interactive effects on RB5 removal were visualized using three-dimensional (3D) and two-dimensional (2D) response plots (Fig. 4). These plots display the relationship between two variables at a time, with the remaining factors held at their zero level. Figure 4A&B depicts RB5 removal that increased with decreasing RB5 concentration, with the highest response occurring at an RB5 concentration between − 0.5 and − 2 and a pH level between − 0.5 and 1.5. Over 80% RB5 decolorization was achieved at an incubation time between − 0.8 and 2 and an inoculum size above 0 (Fig. 4C-F). With an RB5 concentration between − 2 and 1.3, and a pH level between 0 and 2, optimal conditions were reached. The contour plots further confirmed the significant interaction between variables, showing a saddle-shaped pattern that indicates their joint effect on decolorization, which is characteristic of significant interactions [55].

**Fig. 5:**
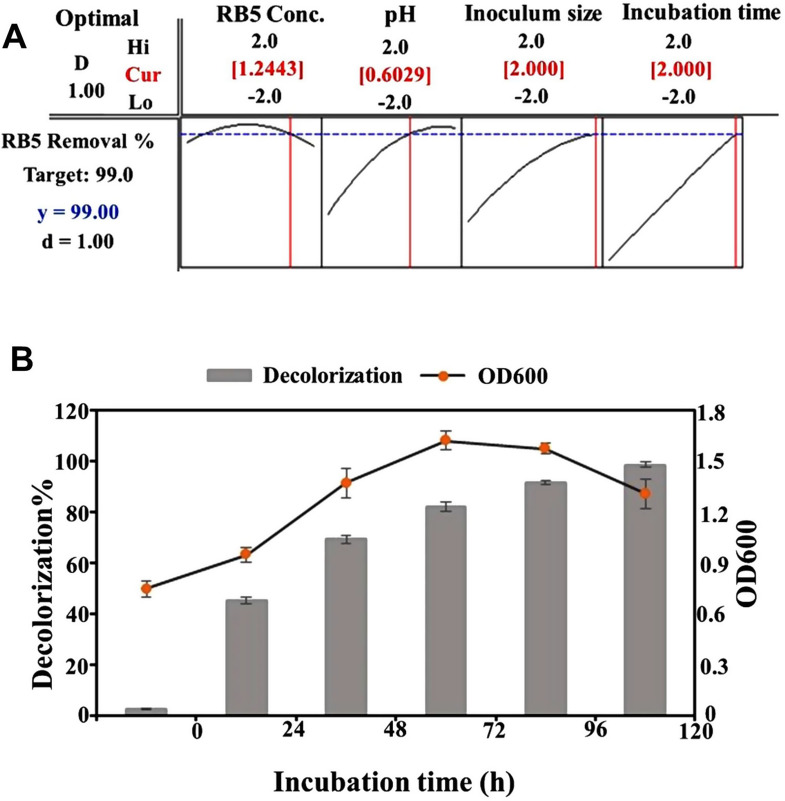
Optimization and validation of RB5 decolorization conditions using statistical modeling and experimental analysis. **(A)** Desirability function plot showing the optimal levels of RB5 concentration (1.24), pH (0.60), inoculum size (2.00), and incubation time (2.00) to achieve the target decolorization efficiency of 99%, with a desirability score (d) of 1.00. **(B)** Experimental validation of the optimized conditions, showing the progression of RB5 decolorization (%) and bacterial growth over a 120-hour incubation period. Maximum decolorization and cell density were observed at 72 h

To predict the optimal conditions for maximum RB5 removal, the optimizer tool was employed, which evaluates individual desirability using a desirability function. The predicted optimal conditions for RB5 decolorization were determined as RB5 concentration (85 mg/L), pH (8.6), incubation time (120 h), and inoculum size (12%) (Fig. 5A). Among the factors influencing RB5 decolorization, pH plays a crucial role in determining the efficiency of the azo dye removal process [56]. The transport of dye particles across the cell membrane and microbial enzymatic activities are significantly affected by the pH of the decolorization medium (Bera and Tank, 2021). An unsuitable pH can alter the structure of enzyme active sites, thereby reducing the enzyme’s ability to bind to the dye molecules, ultimately hindering the dye degradation process [57]. Since reactive azo dyes bind to cotton fibers through addition or substitution mechanisms at alkaline pH, the pH tolerance of decolorizing bacteria is of critical importance [58].

In this study, the biodegradation efficiency of the developed bacterial consortium increased as the pH increased, reaching its highest removal efficiency at alkaline pH 8.6. This result suggests that the tested consortium can serve as an effective bioremediation tool for textile wastewater treatment. At lower pH values, dye cations face competition with H⁺ ions, leading to a decrease in removal efficiency. Conversely, at higher pH values, electrostatic attraction between negatively charged biomass surfaces and positively charged azo dye cations is enhanced, improving decolorization efficiency [59]. The optimal pH for RB5 removal by bacterial consortia StSp and PsGo was 9 and 11, respectively [51]. RB5 decolorization by the halotolerant yeast consortium HYC was most effective at neutral (pH = 7) [4].

Another key factor in the optimization process is the cell-to-dye ratio, which governs the efficiency of azo dye bioremediation. A low bacterial cell density in the presence of a high dye concentration results in lower removal rates [24]. This effect may be due to dye molecules blocking and saturating a limited number of enzyme active sites, thereby reducing degradation efficiency. In contrast, a higher inoculum size promotes bacterial growth and enzyme production, leading to a significant improvement in decolorization efficiency [60]. In this study, the highest decolorization efficiency was observed at an inoculum size of 12% for RB5 (85 mg/L). Decolorization rates of 100 mg/L RB5 and Reactive Red 120 exceeded 90% after 72 h of incubation using a 10% inoculum volume of three bacterial strains [61]. Additionally, *Bacillus albus* DD1 achieved 98% RB5 removal (50 mg/L) at an inoculum size of 25% [49].

However, beyond a certain inoculum size, the decolorization potential began to decline, likely due to nutrient depletion and reduced enzyme production caused by excessive biomass. This leads to a drop in microbial metabolic activity [62]. Additionally, excessively high azo dye concentrations can hinder the decolorization process, likely due to the presence of sulfonic groups on the dye’s aromatic rings, which inhibit microbial growth at higher concentrations [63]. Thus, achieving an optimal balance between dye concentration and inoculum size is essential for maximizing decolorization efficiency. In addition to pH and inoculum size, incubation time was another crucial factor affecting RB5 decolorization. The findings of this study revealed that longer incubation (120 h) yielded the highest RB5 removal efficiency. This result is consistent with previous studies, such as that found by Eskandari et al. [51] who demonstrated that a consortium of mesophilic and cold-adapted bacteria, affiliated to *Pseudoarthrobacter*, *Gordonia*, *Stenotrophomonas*, and *Sphingomonas* genera, decolorized 54% and 34% of RB5 (50 mg/L) within 7 days under static conditions. Whereas, Thakur et al. [64] found that *Bacillus* sp. exhibited optimal decolorization of Red HE7B azo dye after 144 h.

The application of PBD and CCD for RB5 removal optimization successfully predicted the optimal values of the significant factors, leading to the highest decolorization efficiency of RB5 by the bacterial consortium. To validate the optimization model, an experimental validation study was performed under the predicted optimal conditions obtained from CCD. The actual RB5 removal efficiency observed in the experiment was 98.56%, which closely matched the predicted decolorization efficiency of 99% (Fig. 5B). This strong agreement confirms the validity and accuracy of the optimization model. Furthermore, the optimization process resulted in a nearly twofold increase in decolorization efficiency, from 57.21% (before optimization) to 98.56% (after optimization). These results highlight the potential of the optimized bacterial consortium as a highly efficient bioremediation approach for the removal of RB5 and other reactive azo dyes from industrial wastewater. Accordingly, the bacterial consortium evaluated in the current study appears to be a promising and advantageous tool for the efficient remediation of RB5 at a relatively high concentration (85 mg/L) under economically favorable conditions and within an appropriate timeframe (120 h), when compared to previous studies. For example, a halotolerant yeast consortium HYC achieved a decolorization efficiency of 96.1% when treating 50 mg/L of RB5 under static conditions at pH 7.0 and 35 °C within 120 h [4]. Similarly, Seyedi et al. [26] demonstrated that a consortium of three haloalkaliphilic bacterial strains was capable of decolorizing 87% of RB5 and 85% of Reactive Red 152 (both at 50 mg/L) within 120 h under static conditions, noting that the bioremediation process was conducted in nutrient-enriched media supplemented with 5% yeast extract, 10% peptone, and 10% glucose.

### Effect of hypersaline conditions

**Fig. 6:**
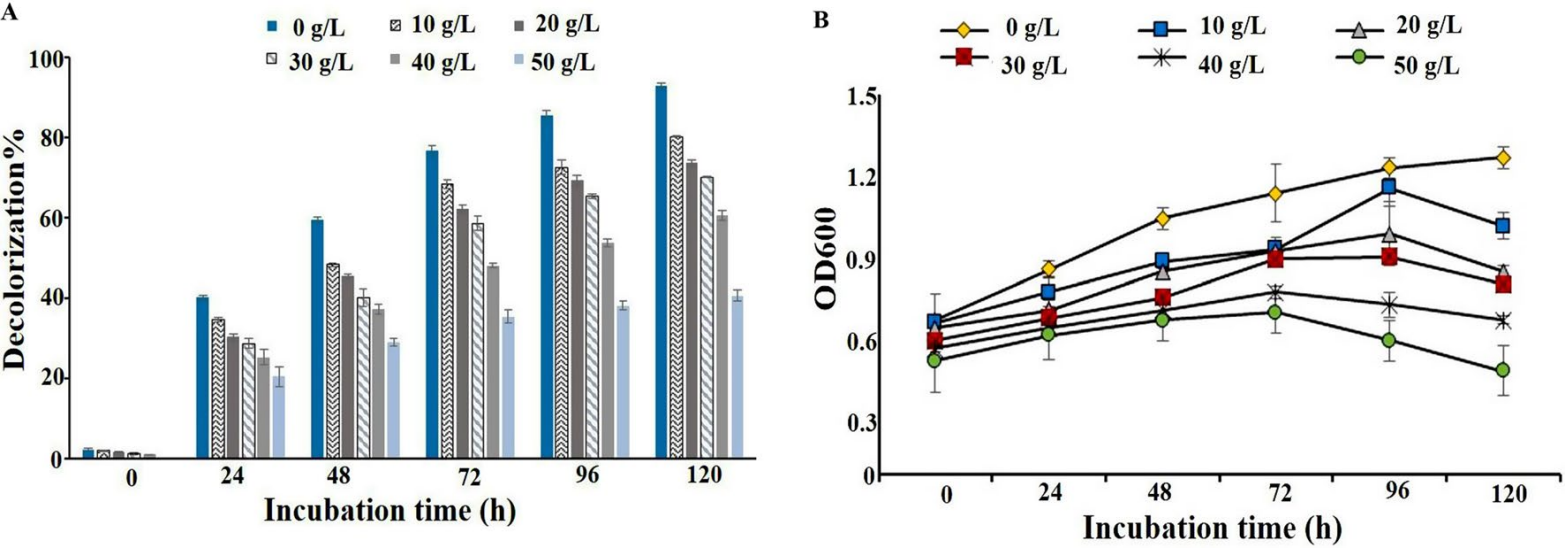
Effect of increasing NaCl concentrations on RB5 decolorization efficiency and bacterial growth over time. **(A)** Decolorization (%) of RB5 by the bacterial consortium at different NaCl concentrations (0–50 g/L) across a 120-hour incubation period. Decolorization efficiency decreased with increasing salt concentration, indicating salt stress negatively impacts dye removal. **(B)** Corresponding growth patterns of the bacterial consortium under the same NaCl concentrations, showing reduced growth at higher salt levels, particularly at 40 and 50 g/L

Microbial activity and survival are significantly affected by high salinity, which poses a major challenge in the microbial remediation of textile wastewater effluents. Many naturally occurring bacteria are highly sensitive to salinity stress, limiting their ability to efficiently degrade contaminants in saline environments [18]. In this study, the impact of high NaCl concentrations on RB5 decolorization efficiency and the growth rate of the bacterial consortium was investigated, as illustrated in Fig. 6. The bacterial consortium exhibited high decolorization efficiency, ranging between 92.76% and 70.06% across NaCl concentrations of 0–30 g/L after 120 h of incubation. However, when the salinity level increased further to 40 g/L and 50 g/L, decolorization efficiency dropped significantly to 60.45% and 40.61%, respectively. The observed decrease in RB5 decolorization at elevated NaCl concentrations can be attributed to multiple factors. One major factor is the reduction in RB5 dye solubility, which can limit microbial access to the dye molecules. Additionally, high salt concentrations have been shown to inhibit microbial enzymatic activities, sometimes leading to complete enzyme inactivation [65]. Saline environments can also induce plasmolysis in bacterial cells, leading to loss of cellular activity and decreased metabolic efficiency. Given the challenges associated with salinity stress, the isolation of halophilic or halotolerant bacterial strains is crucial for improving the treatment of textile effluents containing both azo dyes and high salt concentrations. In this study, the bacterial consortium demonstrated a high growth rate at NaCl concentrations ranging from 10 to 30 g/L, suggesting that the developed consortium is salt-tolerant and can effectively function under moderately saline conditions. This property makes the consortium a promising and economically viable bioremediation tool for the treatment of saline textile wastewater, which is often encountered in industrial effluents.

### Enzymatic activities of developed bacterial consortium

The bacterial consortium demonstrated efficient RB5 degradation, which can be attributed to its unique enzymatic system. To elucidate the RB5 decolorization mechanism, the activities of intracellular oxidases (Lac, MnP, LiP) and reductases (azoreductase and NADH-DCIP reductase) were quantified (Table 7). Compared to the control, the activity of all tested enzymes in the cell extracts significantly increased, suggesting that the presence of RB5 azo dye induced the expression of key enzymes involved in the degradation process. Among the analyzed enzymes, NADH-DCIP reductase exhibited the highest activity (32.14 ± 0.07 U/mg), followed by azoreductase (20.23 ± 0.015 U/mg). In contrast, the activities of oxidative enzymes were relatively lower, with LiP (4.59 ± 0.36 U/mg), Lac (2.52 ± 0.017 U/mg), and MnP (0.081 ± 0.002 U/mg). The strong activity of reductive enzymes suggests that the bacterial consortium primarily follows a reductive degradation pathway, which may also explain the low decolorization efficiency under shaking conditions. These findings are consistent with those of Bera and Tank [66], who reported that *Pseudomonas stutzeri* SPM-1 exhibited high azoreductase and NADH-DCIP reductase activity during the biodegradation of Procion Red H3B azo dye.

**Table 7:** Intracellular enzyme activities of tested bacterial consortium for degradation of 85 mg/l of RB5

Enzyme	Unit	Initial activity at 0 h	Final activity at the end of degradation
Azoreductase	µM of methyl red reduced per min per mg protein	1.62 ± 0.011	20.23 ± 0.015***
Laccase	Units per min per mg protein	0.114 ± 0.002	2.52 ± 0.017***
Lignin peroxidase	Units per min per mg protein	0.183 ± 0.004	4.59 ± 0.36***
Mn peroxidase	Units per min per mg protein	0.034 ± 0.003	0.081 ± 0.002**
NADH-DCIP reductase	µM of DCIP reduced per min per mg protein	9.58 ± 0.496	32.14 ± 0.07***

Values are mean ± S.D of triplicate measurements of the enzyme activity;

*, **, ***significantly different from control (0 h) at *P* < 0.05, *P* < 0.01, and *P* < 0.001, respectively, using one-way analysis of variance (ANOVA)

Several studies have emphasized the key role of NADH-DCIP reductase and azoreductase in the reductive cleavage of azo bonds, leading to the breakdown of azo dyes into intermediate aromatic amines [45, 67]. In the present study, the moderate production of oxidative enzymes suggests that these intermediates were further oxidized into smaller and non-toxic compounds, highlighting the synergistic role of reductive and oxidative enzymes in the degradation and detoxification of RB5 azo dye. To gain further insights into the RB5 biodegradation process, the degradation products were analyzed using UV–visible spectroscopy, FTIR spectroscopy, and GC–MS. These analyses aimed to identify the breakdown products and confirm the complete mineralization of RB5 into non-toxic metabolites.

### Metabolites characterization

**Fig. 7:**
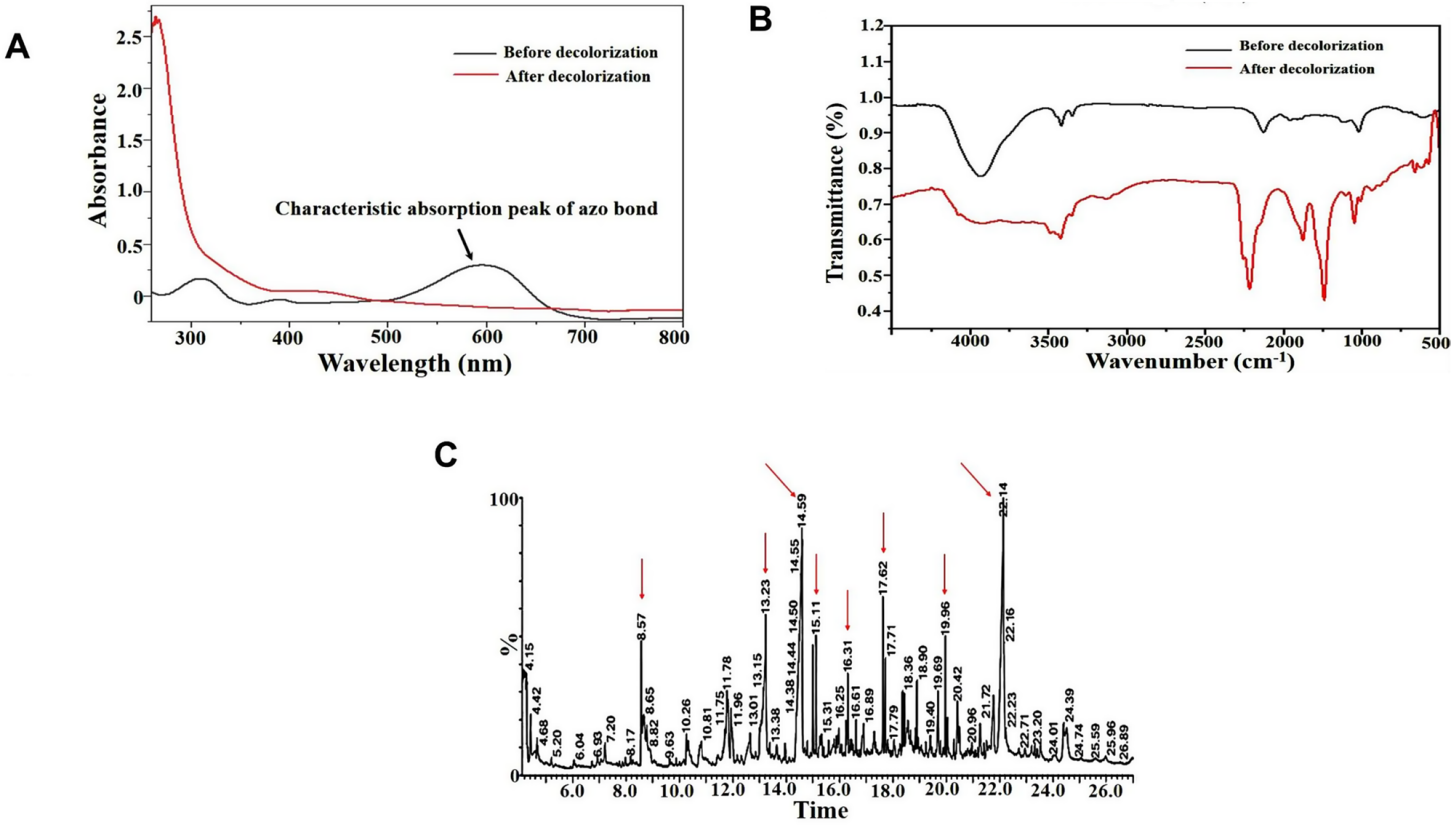
A Spectroscopic and chromatographic analysis of RB5 dye before and after microbial decolorization. **(A)** UV–Vis spectra showing the disappearance of the characteristic azo bond absorption peak (~ 597 nm) after decolorization, indicating successful dye degradation. **(B)** FTIR spectra illustrating major changes in transmittance patterns, suggesting breakdown of functional groups and structural modifications post-decolorization. **(C)** GC-MS chromatogram of the dye sample after decolorization, showing multiple peaks corresponding to degraded metabolites, indicating the dye’s transformation into simpler compounds

The UV-Vis spectral analysis of RB5 dye revealed a maximum absorption peak at 597 nm, which is characteristic of aromatic rings attached to the azo bond. Additionally, a secondary peak at 310 nm was observed, corresponding to benzene-like structures. Upon complete decolorization of RB5, the characteristic peak at 597 nm disappeared entirely, confirming the cleavage of azo bonds by the bacterial consortium under optimal conditions (Fig. 7A). Similar results have been reported by Srivastava et al. [49] and Emadi et al. [17], who observed a sharp decline in the intensity of RB5’s characteristic peaks after bacterial decolorization. The observed spectral changes before and after biodegradation indicated significant structural modifications in the RB5 molecule, as further confirmed by the identification of degradation metabolites [4].

The FTIR analysis provided additional evidence of structural modifications in the RB5 molecule following degradation by the bacterial consortium (Fig. 7B). The control RB5 spectrum exhibited a prominent peak at 3435 cm⁻¹, which corresponds to–NH and O–H stretching. Peaks at 2924 cm⁻¹ and 2846 cm⁻¹ were associated with CH₂ stretching, while a peak at 1614 cm⁻¹ was assigned to the N = N stretching of the azo bond, which is primarily responsible for the color of the dye. Additionally, peaks in the 1021–1126 cm⁻¹ range corresponded to C–O stretching, whereas the sulfone SO₂ stretching was observed at 1126 cm⁻¹. The presence of secondary amines was also indicated by a peak at 609 cm⁻¹ [28, 29]. Following RB5 biodegradation, the azo bond absorption at 1614 cm⁻¹ disappeared, confirming the cleavage of the azo linkage. Furthermore, the appearance of new peaks at 3490, 1715, 1400, and 567–879 cm⁻¹ was observed, corresponding to O–H, C = O, C–C, and C–N stretching vibrations, respectively. These structural changes suggest the elimination of amines from the biotransformation product [68]. The observed differences between the control and degraded RB5 spectra confirm the successful biodegradation of RB5 by the bacterial consortium, which facilitated the breakdown of complex dye molecules into smaller, less toxic intermediates.

To further elucidate the biodegradation pathway, the extracted metabolites were subjected to GC-MS analysis, which identified multiple degradation products (Table 8). As depicted in Fig. 7C, the GC-MS chromatogram displayed major peaks at retention times 8.57, 13.23, 14.59, 15.11, 16.31, 17.62, 19.96, and 22.14 min, which were identified as Benzoic acid (m/z 122), 2-Octene (m/z 112), Phthalic anhydride (m/z 148), Naphthalene (m/z 128), Isopropyl-dimethyl-hexahydro-naphthalene (m/z 204), 1-Sulphonic,2-(4-aminobenzenesulphonyl) ethanol (m/z 280), n-Hexadecanoic acid (m/z 256), and Ocadecenoic acid (m/z 284). The biodegradation of reactive azo dyes generally proceeds through symmetrical or asymmetrical cleavage of azo bonds [69]. The reductive cleavage of azo bonds by azoreductase and NADH-DCIP reductase is typically the first step in azo dye biodegradation [7]. Based on the identified intermediates, the bacterial consortium asymmetrically cleaved RB5, predominantly catalyzed by NADH-DCIP reductase and azoreductase, leading to the formation of the aromatic amine intermediate 1-sulphonic,2-(4-aminobenzenesulphonyl) ethanol (m/z 280).

**Table 8:** RB5 degradation products identified by GC-MS

Compound	m/z	Molecular formula	Retention time (minute)
Benzoic acid	122	C_7_H_6_O	8.57
2-octene	112	C_8_H_16_	13.23
Phthalic anhydride	148	C_8_H_4_O_3_	14.59
Naphthalene	128	C_10_H_8_	15.11
Isopropyl-dimethyl-hexahydro-naphthalene	204	C_15_H_24_	16.31
1-sulphonic,2-(4-aminobenzenesulphonyl) ethanol	280	C_8_H_11_NO_6_S_2_	17.62
n-Hexadecanoic acid	256	C_16_H_32_O_2_	19.96
Octadecenoic acid	284	C_18_H_34_O_2_	22.14

**Fig. 8:**
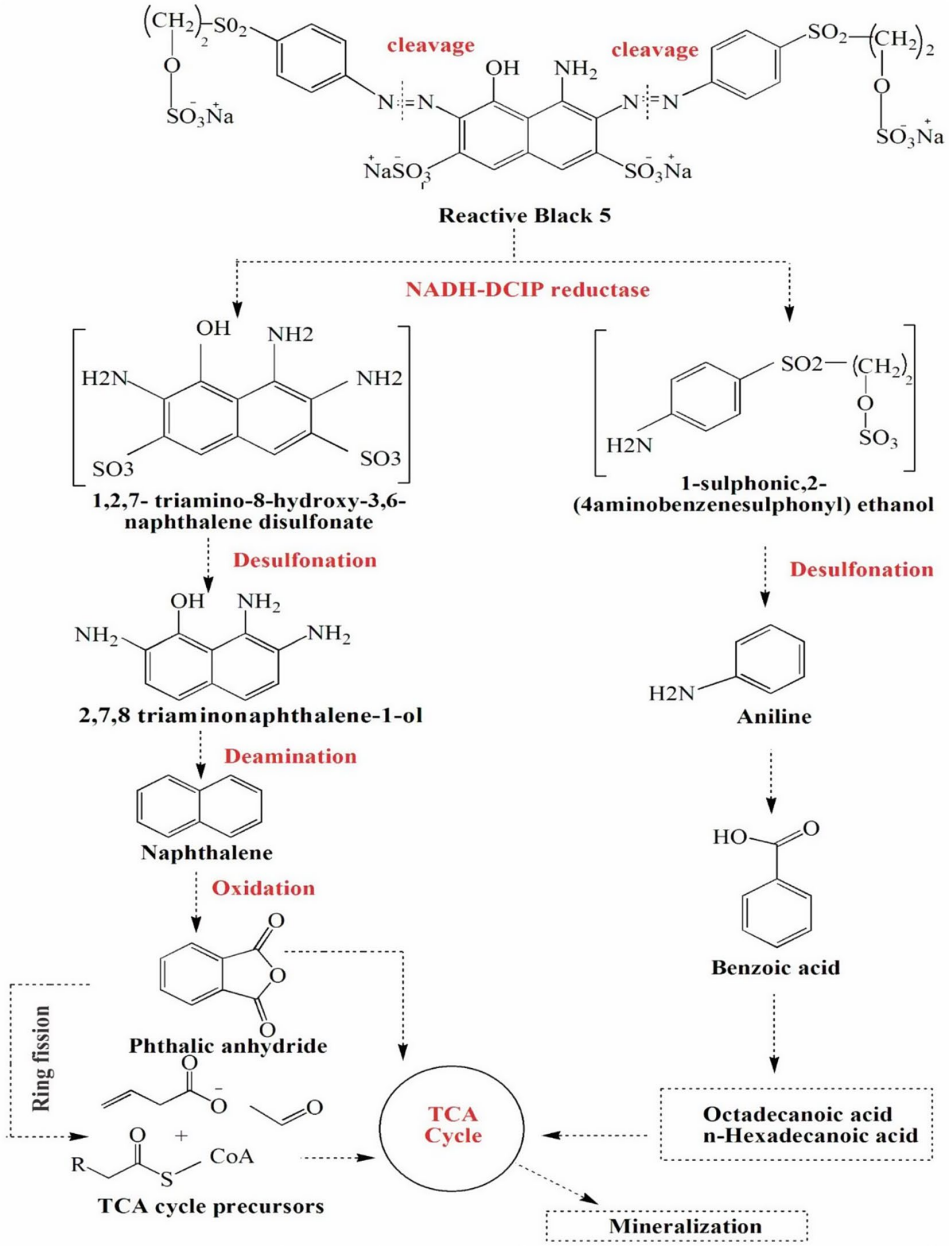
Proposed metabolic pathway for the microbial degradation of RB5. The initial azo bond cleavage is catalyzed by NADH-DCIP reductase, producing aromatic amines including 1,2,7-triamino-8-hydroxy-3,6-naphthalene disulfonate and 1-sulphonic-2-(4-aminobenzenesulphonyl) ethanol. Subsequent desulfonation, deamination, and oxidation steps lead to the formation of simpler intermediates such as aniline, naphthalene, benzoic acid, and phthalic anhydride. These are further metabolized through ring fission into TCA cycle precursors, eventually resulting in complete mineralization into CO₂ and H₂O

Interestingly, another common amine intermediate, 1,2,7-triamino-8-hydroxy-3,6-naphthalinedisulfonate (TAHNDS), was not detected. This could be due to its instability and rapid breakdown into smaller compounds [70]. The presence of naphthalene (m/z 128) and its derivatives (m/z 204) suggests that desulfonation and deamination reactions facilitated the breakdown of TAHNDS into less complex aromatic structures. Ultimately, the degradation products of 1-sulphonic,2-(4-aminobenzenesulphonyl) ethanol and TAHNDS were further oxidized into smaller, less toxic aliphatic compounds. Several studies have shown that amines generated during azo dye biodegradation can be further decomposed into low-molecular-weight compounds and eventually mineralized [34, 71]. The proposed biodegradation mechanism of RB5 by the bacterial consortium involves the breakdown of azo bonds (N = N), which are responsible for the chromophoric properties of the dye (Fig. 8). These findings were corroborated by the UV-Vis and FTIR analyses, which provided strong spectroscopic evidence of azo bond cleavage. However, further analytical studies are necessary to fully confirm the biodegradation pathway at a molecular level.

The biotransformation intermediates detected in this study, including octadecenoic acid, n-hexadecanoic acid, 2-octene, and benzoic acid, demonstrate the ability of the bacterial consortium to degrade RB5 into non-toxic compounds. Benzoic acid is particularly significant, as it serves as a precursor for fatty acid synthesis during the azo dye biodegradation pathway [72]. Furthermore, previous studies have indicated that octadecenoic acid and n-hexadecanoic acid are common fatty acids formed during degradation [28]. Additionally, octadecenoic acid and n-hexadecanoic acid exhibit antimicrobial, anti-inflammatory, antioxidant, and cancer-preventive properties [73, 74]. These findings suggest that the biodegradation of RB5 not only removes toxic azo dyes from wastewater but may also yield biologically valuable metabolites. The high detoxification efficiency of the developed consortium can be attributed not only to the individual capabilities of the bacterial strains but also to their cooperative metabolic interactions. Within the consortium, *Bacillus cereus* likely contributes robust enzyme production and biofilm formation, *Proteus mirabilis* may enhance substrate uptake and tolerance to high dye concentrations, while *Stenotrophomonas maltophilia* may support oxidative degradation and resilience in saline environments. This division of labor ensures that multiple enzymatic pathways — both reductive and oxidative — operate in parallel, enabling efficient breakdown of complex dye molecules. The metabolic versatility of the consortium facilitates sequential and complementary degradation steps, such as azo bond cleavage followed by aromatic ring oxidation, leading to non-toxic metabolites [7]. Such synergy reflects natural microbial consortia behavior where niche specialization and resource sharing increase ecological fitness and biodegradation capacity. Future studies using transcriptomics or metabolic flux analysis could further elucidate the molecular basis of this cooperative detoxification mechanism.

The results of UV-Vis, FTIR, and GC-MS analyses provide compelling evidence that RB5 degradation by the developed bacterial consortium proceeds through a reductive pathway, followed by further oxidation into smaller, less toxic compounds. Among the detected RB5 degradation products, phthalic anhydride and naphthalene are noteworthy due to their established roles as environmental pollutants. Phthalic anhydride, while commonly used in plasticizers and resins, is known to be an irritant and has been reported to cause developmental toxicity in aquatic species upon prolonged exposure [75]. Similarly, naphthalene, a polycyclic aromatic hydrocarbon, is recognized for its potential toxicity, bioaccumulation, and carcinogenicity, particularly in aquatic environments. However, in this study, both compounds were detected only as intermediate metabolites, and their concentrations likely remained low due to further downstream oxidation and conversion into less toxic aliphatic compounds, such as octadecenoic acid and benzoic acid (Fig. 8). This transformation is supported by GC-MS profiling and the observed decline in toxicity in phytotoxicity, biotoxicity, and cytotoxicity assays. These findings suggest that although transient ecotoxic compounds may arise during dye degradation, the bacterial consortium ultimately facilitates their breakdown, resulting in a significantly less toxic metabolite profile​. The identified intermediates confirm that the consortium effectively mineralizes RB5, making it a promising bioremediation tool for textile wastewater treatment.

### Phytotoxicity assessment

**Fig. 9:**
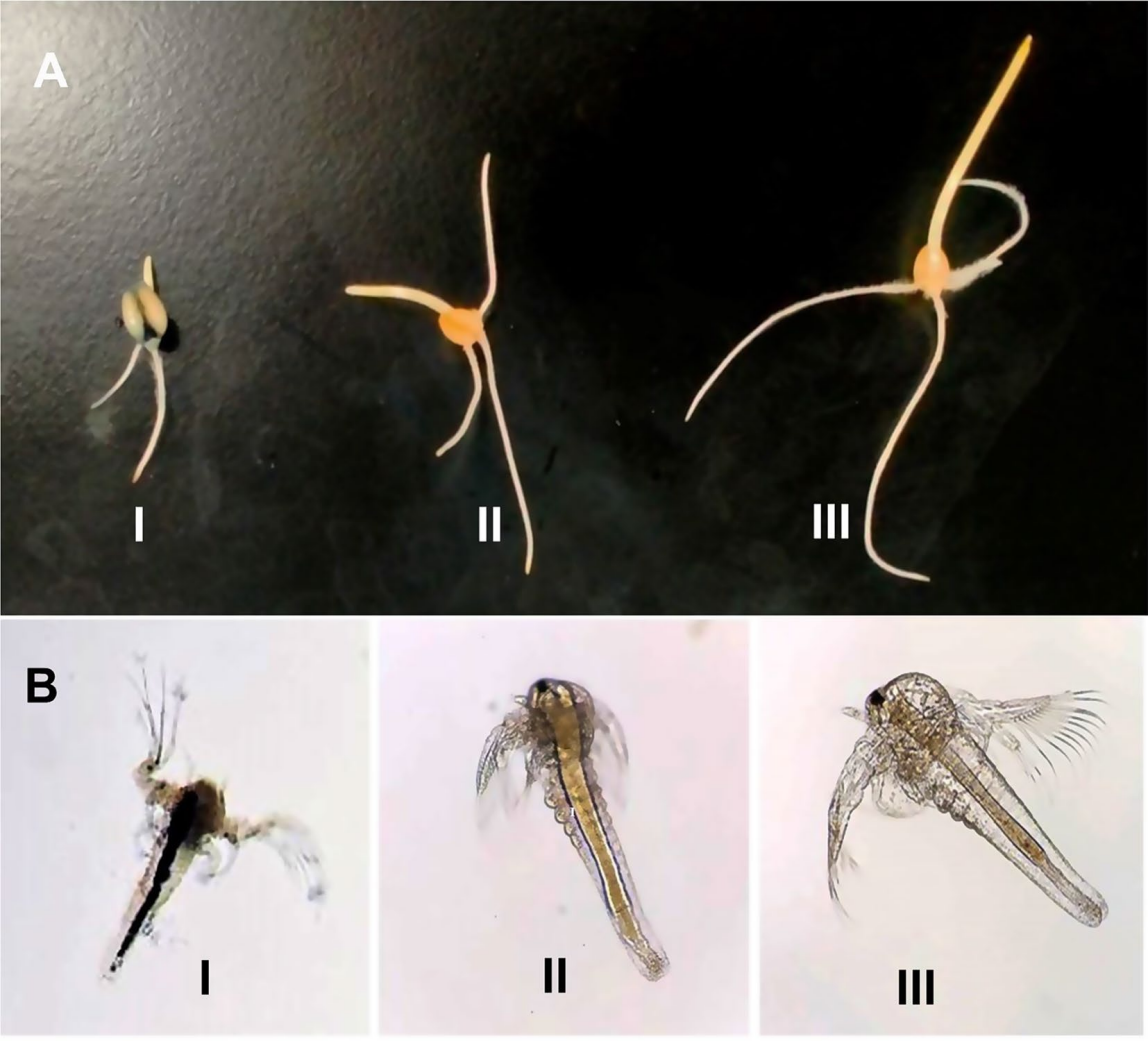
Evaluation of phytotoxicity and biotoxicity of untreated and treated RB5 dye. **(A)** Phytotoxicity assessment on *Triticum aestivum* (wheat) seeds. (I) Seeds treated with untreated RB5 showed inhibited growth. (II) Seeds treated with treated RB5 (biodegraded metabolites) exhibited moderate growth. (III) Seeds irrigated with distilled water (control) showed normal and healthy growth. **(B)** Biotoxicity assessment using *Artemia salina*. (I) Accumulation of untreated RB5 in the mid-gut of *A. salina*. (II) Accumulation of degraded RB5 metabolites in the mid-gut of *A. salina*. (III) *A. salina* exposed to artificial seawater (control), showing normal morphology

Azo dyes and other toxic pollutants have been shown to inhibit plant hormone activity, particularly phytohormones, which play a crucial role in seed germination [76]. Furthermore, the biodegradation of azo dyes may lead to the release of hazardous amine compounds, which can have detrimental environmental effects. Therefore, toxicity studies are essential to determine the suitability of treated wastewater for discharge into water bodies and potential agricultural applications, particularly in regions facing water scarcity [77]. In this study, acute phytotoxicity tests revealed a significant reduction in root growth inhibition from approximately 66.38% in untreated dye solutions to 21.38% following the treatment process. Additionally, *T. aestivum* seeds treated with degraded RB5 metabolites exhibited a high germination rate of 93.33%, compared to a low germination rate of 40% when exposed to untreated RB5 dye (Table 9; Fig. 9A). These findings confirm that RB5 azo dye was efficiently degraded by the bacterial consortium, resulting in significantly less toxic degradation metabolites.

**Table 9:** Phytotoxicity test of RB5 dye and its degraded metabolites using seeds of *Triticum aestivuma*

Test solution	Mean root growth (cm)	Mean shoot growth (cm)	Inhibition (%)	Germination (%)
Distilled water	3.60 ± 0.36	2.80 ± 0.10	NI	100
Metabolites	2.93 ± 0.15 *	2.46 ± 0.25	18.61	93.33
Untreated RB5	1.21 ± 0.22 ***	0.933 ± 0.51**	66.38	40

NI, no inhibition

Values are presented as the mean ± SD of triplicate measurements,

*, **, ***significantly different from control (distilled water) at *P* < 0.05, *P* < 0.01, and *P* < 0.001, respectively, using one-way analysis of variance (ANOVA)

The influence of RB5 biodegradation products on plant growth and seed germination has been previously documented in various studies. RB5 treated by *Bacillus cereus* ROC significantly increased the seed germination rate of *Solanum lycopersicum* to 86.5% and 85.8%, compared to only 13% and 6.1% germination rates in untreated dye solutions at 150 and 250 mg/L, respectively [78]. Similarly, it has been reported that treated RB5 solutions using *Phanerochaete chrysosporium* exhibited higher germination rates (70% and 63%) for *Lactuca sativa* compared to 50% and 37% for untreated RB5 solutions at concentrations of 50 and 100 mg/L, respectively [79]. However, RB5 biologically treated by *Bacillus cereus* MS038EH exhibited phytotoxic effects on *T. aestivum* and maize seeds due to the formation of intermediate toxic amines [17]. These discrepancies highlight the importance of further toxicity assessments to ensure that biodegradation metabolites are completely detoxified before wastewater discharge or reuse in agriculture. The results of this study suggest that the developed bacterial consortium successfully degraded RB5 into less toxic compounds, making the treated wastewater potentially suitable for agricultural irrigation. However, further environmental impact studies and long-term assessments of biodegradation metabolites are recommended to ensure the complete safety of treated effluents before their application in irrigation systems or discharge into aquatic environments.

### Biotoxicity study

*Artemia salina* has been widely used as a bioindicator for assessing the toxicity of textile wastewater, as effluents from textile industries are often characterized by high salinity and conductivity, which are critical factors affecting aquatic species. The biotoxicity assay conducted in this study demonstrated that untreated RB5 dye exhibited an average mortality rate of 86.7%, whereas the degraded RB5 metabolites resulted in a significantly lower mortality rate of 23.3%. The substantial decrease in brine shrimp mortality indicates that the RB5 dye was effectively detoxified following treatment by the bacterial consortium. The survival rate exceeding 50% in brine shrimp exposed to degraded RB5 metabolites further supports the lower toxicity of the degradation products (Table 10). Visual observations further confirmed the toxicity of untreated RB5 dye and its detoxification following treatment. *Artemia salina* exposed to untreated RB5 dye exhibited dark coloration in the gut region, indicating dye accumulation within the organism. In contrast, *Artemia salina* exposed to the degraded RB5 dye showed only a light-colored substance deposited in the mid-gut region, suggesting that the breakdown products of RB5 are less toxic and accumulate at lower concentrations [80]. Furthermore, no significant color changes were observed in the gut region of brine shrimp exposed to artificial seawater alone, which was used as a control group (Fig. 9B). These findings reinforce the effectiveness of the bacterial consortium in reducing the toxicity of RB5, making the treated effluent safer for aquatic organisms.

**Table 10:** Biotoxicity test of RB5 and its biodegradation by-products on *Artemia Salina*

Test solution	No. of dead nauplii	Mortality (%)
Distilled water	0.0 ± 0.0	NM
Metabolites	2.33 ± 0.5**	23.3
Untreated RB5	8.66 ± 1.15***	86.7

NM, no mortality

Values are presented as the mean ± SD of triplicate measurements,

*, **, ***significantly different from control (distilled water) at *P* < 0.05, *P* < 0.01, and *P* < 0.001, respectively, using one-way analysis of variance (ANOVA)

### *In- vitro* cytotoxicity of RB5 and its metabolites on normal human cell lines

**Fig. 10:**
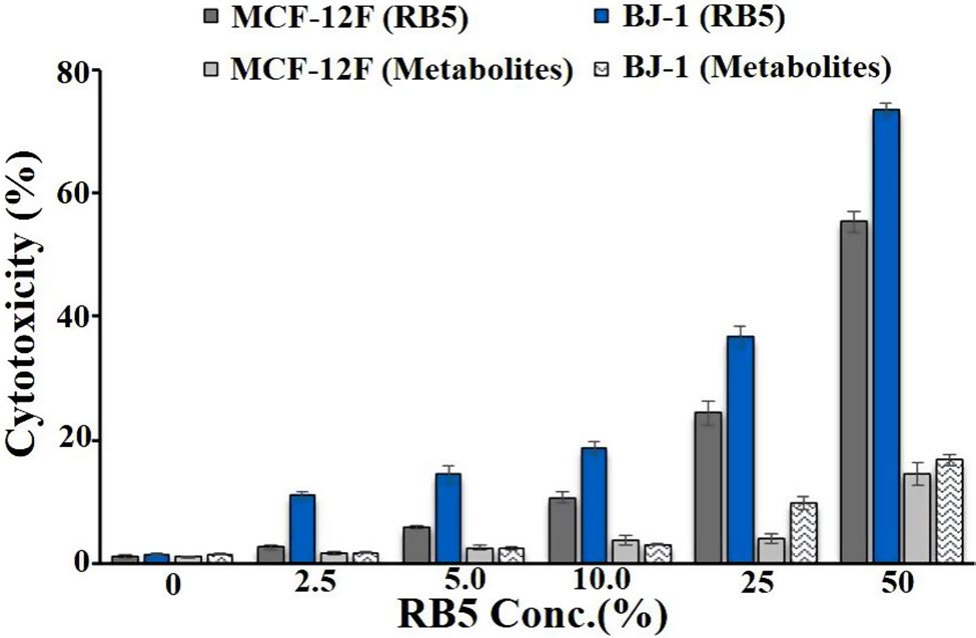
Cytotoxicity assessment of untreated Reactive Black 5 (RB5) and its biodegraded metabolites on human cell lines. The bar graph compares cytotoxic effects of increasing concentrations of RB5 and its metabolites on MCF-12 F (normal human mammary epithelial cells) and BJ-1 (normal human fibroblasts). Untreated RB5 shows dose-dependent cytotoxicity, especially in BJ-1 cells, whereas the metabolites exhibit significantly reduced toxicity across all concentrations, indicating effective detoxification post-biodegradation

The MTT assay is a well-established method for evaluating cellular responses to toxic substances, providing valuable insights into cell survival and death rates in response to potential toxins [40]. In this study, the cytotoxicity of untreated RB5 dye and its degraded metabolites was assessed on two normal human cell lines. The results revealed that the degraded metabolites exhibited significantly lower cytotoxicity compared to the original dye solution, even at the highest tested concentration (50%). At this concentration, the treated dye solution showed cytotoxicity of only 14.45% in MCF-12 F and 16.87% in BJ-1, whereas the untreated RB5 dye exhibited cytotoxicity rates of 55.31% and 73.45%, respectively. These findings confirm that RB5 was effectively degraded by the bacterial consortium into less toxic metabolites, reducing its harmful effects on human cells. Additionally, a dose-dependent cytotoxic response was observed, where cell viability decreased (i.e., cell inhibition increased) as the concentration of both untreated RB5 dye and its degradation products increased. However, even at the highest concentration of degraded metabolites, the cytotoxic effect did not reach IC_50_, indicating that the transformed metabolites were significantly less toxic than the parent dye. In contrast, untreated RB5 dye exhibited IC_50_ values of 46.30% for MCF-12 F and 33.21% for BJ-1, indicating higher toxicity levels (Fig. 10). These findings align with the results reported by Alam et al. [81], who investigated the cytotoxic effects of three azo dyes (RB5, Acid Blue 113, and Acid Orange 7) on human keratinocyte (HaCaT) and human dermal fibroblast (HDF) cells. Their study found that the cytotoxicity of these dyes was reduced by approximately 25% following treatment with the white-rot fungus *Trametes hirsuta* D7, compared to untreated azo dyes, which had a significantly toxic impact on the tested cell lines (Table S3).

While the current study demonstrated effective RB5 decolorization and detoxification in a controlled synthetic medium, future work should focus on evaluating the consortium’s performance in real textile wastewater. This would involve monitoring reductions in key environmental indicators such as biochemical oxygen demand (BOD), chemical oxygen demand (COD), total suspended solids (TSS), total dissolved solids (TDS), and visible color intensity, to assess overall treatment efficacy under practical conditions. Similar approaches have been successfully implemented in recent work by Maniyam et al. [82], where biodecolorization of malachite green was validated using real wastewater matrices. Applying such comprehensive assessments will provide deeper insight into the consortium’s scalability and environmental compliance for industrial applications. Overall, the results of this study confirm that RB5 decolorization by the bacterial consortium resulted in a significant reduction in toxicity, making the treated wastewater potentially safer for discharge and reuse in environmental applications. However, further long-term toxicity studies and comprehensive metabolite profiling are necessary to ensure that the degraded metabolites are entirely non-toxic before large-scale implementation of this bioremediation approach.

While GC-MS analysis identified several intermediate metabolites, including phthalic anhydride, naphthalene derivatives, and benzoic acid, individual toxicity assessments of these compounds were not performed. Instead, the study focused on evaluating the overall toxicity of the treated effluent through a combination of phytotoxicity, biotoxicity, and cytotoxicity assays. These assays showed a significant reduction in toxicity after RB5 degradation, suggesting that the harmful effects of intermediates were either minimal or further mitigated by downstream metabolism. However, future studies involving isolated metabolite standards or metabolomics-guided toxicity screening would be valuable for confirming the safety of individual degradation products.

## Conclusion

This study presents a comprehensive and integrative approach to the biodegradation of the azo dye RB5 using a halotolerant bacterial consortium composed of *Bacillus cereus*, *Proteus mirabilis*, and *Stenotrophomonas maltophilia*. Unlike earlier studies that relied on single strains or focused solely on decolorization, this work employed statistical optimization (PBD and CCD), enzymatic profiling, and multi-tier toxicity analysis to ensure both efficiency and safety of the bioremediation process. The optimized consortium achieved a high decolorization efficiency of 98.56% under static, saline conditions. Enzymatic assays confirmed the role of key reductive enzymes such as azoreductase and NADH-DCIP reductase in azo bond cleavage, while oxidative enzymes further supported downstream degradation. Metabolite analysis using FTIR and GC-MS revealed the breakdown of RB5 into less harmful compounds. Moreover, toxicity assessments demonstrated significant improvements, including reductions in phytotoxicity (e.g., root inhibition reduced from 66.38 to 21.38%), *Artemia salina* mortality (from 86.7 to 23.3%), and cytotoxicity in human epithelial cells (from 55.31 to 14.45%). This multifaceted study addresses key limitations in previous RB5 remediation efforts by demonstrating not only high decolorization efficiency but also mechanistic understanding, statistical process optimization, and validated safety through multi-level toxicity reduction. These combined contributions distinguish this work from earlier studies and advance the scientific basis for real-world implementation. While this study focused on decolorization and detoxification in a controlled laboratory setting using synthetic media, we recognize that actual textile effluents present more complex and challenging conditions, particularly high alkalinity (pH > 10), salinity, and the presence of heavy metals or surfactants. The current optimization identified pH 8.6 as ideal for bacterial activity, which does not fully reflect the highly alkaline nature of untreated textile wastewater. Although the consortium exhibited salt tolerance and strong detoxification potential based on phytotoxicity, biotoxicity, and cytotoxicity assays, further investigation is needed to assess its robustness under industrial conditions. In future work, we plan to evaluate the consortium in simulated or real textile wastewater, monitor performance under extreme pH, and analyze the impact of heavy metals and co-contaminants on decolorization and survival. These studies will be critical to validating the system for full-scale application in industrial wastewater treatment. Future studies should focus on the consortium’s application to real textile wastewater and monitor reductions in physicochemical parameters such as BOD, COD, TSS, TDS, and color. Additional research is also needed to evaluate system performance under variable environmental conditions, explore long-term stability, and assess scalability for industrial use. These steps will strengthen the case for regulatory approval and support the deployment of biologically-based solutions in alignment with the UN Sustainable Development Goals (SDGs), particularly SDGs 6, 12, and 13.

## Electronic supplementary material

Below is the link to the electronic supplementary material.

Supplementary Material 1

## Data Availability

No datasets were generated or analysed during the current study.
