# Supplementary material for: Efficient biodegradation and detoxification of reactive black 5 using a newly constructed bacterial consortium
Source: Microb Cell Fact. 2025 Jul 2;24:154. doi: 10.1186/s12934-025-02768-z (PMC12218093; doi:10.1186/s12934-025-02768-z)
Supplement: Supplementary file 1 — Supplementary Material 1 [file 12934_2025_2768_MOESM1_ESM.docx]

**Table S1.** PBD matrix of independent variables with experimental and predicted results.

| **Run** | **RB5 concentration** | **Glucose** | **(NH_4_)_2_SO_4_** | **MgSO_4_** | **NaCl** | **CaCl_2_** | **K_2_HPO_4_** | **KH_2_PO_4_** | **pH** | **Temp** | **Inoculum size** | **Incubation time** | **Decolorization %** | |
| --- | --- | --- | --- | --- | --- | --- | --- | --- | --- | --- | --- | --- | --- | --- |
|  |  |  |  |  |  |  |  |  |  |  |  |  | **Experimental** | **Predicted** |
| **1** | 1 | -1 | 1 | -1 | 1 | -1 | -1 | 1 | -1 | -1 | 1 | -1 | 17.06±0.16 | 20.61 |
| **2** | 1 | 1 | -1 | 1 | -1 | -1 | 1 | 1 | -1 | 1 | -1 | -1 | 6.78±0.22 | 5.38 |
| **3** | -1 | 1 | 1 | 1 | 1 | -1 | 1 | 1 | -1 | -1 | 1 | 1 | 75.04±0.06 | 83.44 |
| **4** | 1 | 1 | 1 | -1 | -1 | 1 | -1 | 1 | 1 | 1 | -1 | -1 | 12.93±0.02 | 16.56 |
| **5** | -1 | -1 | 1 | 1 | -1 | 1 | 1 | 1 | 1 | -1 | -1 | -1 | 70.02±0.17 | 64.49 |
| **6** | 1 | 1 | -1 | 1 | 1 | -1 | -1 | -1 | 1 | -1 | -1 | 1 | 43.13±0.11 | 48.41 |
| **7** | -1 | 1 | -1 | -1 | 1 | 1 | 1 | 1 | -1 | 1 | -1 | 1 | 69.03±0.06 | 63.73 |
| **8** | -1 | 1 | -1 | 1 | 1 | 1 | -1 | -1 | 1 | 1 | 1 | -1 | 88.0±1.0 | 90.22 |
| **9** | -1 | 1 | 1 | -1 | -1 | -1 | 1 | -1 | 1 | -1 | -1 | 1 | 87.56±0.46 | 89.71 |
| **10** | 1 | -1 | -1 | -1 | -1 | -1 | 1 | -1 | 1 | 1 | 1 | 1 | 70.14±0.61 | 79.06 |
| **11** | 1 | 1 | 1 | -1 | 1 | 1 | 1 | -1 | 1 | -1 | 1 | -1 | 74.01±1.01 | 63.76 |
| **12** | -1 | 1 | 1 | 1 | -1 | -1 | -1 | -1 | -1 | 1 | 1 | -1 | 69.52±0.89 | 63.73 |
| **13** | -1 | -1 | 1 | -1 | 1 | 1 | -1 | -1 | -1 | 1 | -1 | 1 | 65.32±0.38 | 66.72 |
| **14** | -1 | -1 | -1 | 1 | -1 | 1 | -1 | 1 | 1 | -1 | 1 | 1 | 86.74±0.95 | 88.37 |
| **15** | -1 | -1 | -1 | -1 | 1 | -1 | 1 | 1 | 1 | 1 | 1 | -1 | 90.0±1.0 | 93.88 |
| **16** | 1 | -1 | 1 | 1 | 1 | -1 | -1 | 1 | 1 | 1 | -1 | 1 | 66.68±1.34 | 54.75 |
| **17** | 1 | -1 | 1 | 1 | -1 | 1 | 1 | -1 | -1 | 1 | 1 | 1 | 50.69±2.04 | 57.07 |
| **18** | 1 | 1 | -1 | -1 | -1 | 1 | -1 | 1 | -1 | -1 | 1 | 1 | 32.77±1.10 | 25.78 |
| **19** | -1 | -1 | -1 | -1 | -1 | -1 | -1 | -1 | -1 | -1 | -1 | -1 | 29.73±0.50 | 28.67 |
| **20** | 1 | -1 | -1 | 1 | 1 | 1 | 1 | -1 | -1 | -1 | -1 | -1 | 9.43±1.50 | 12.19 |

Experimental values are mean ± SD of triplicate measurements.

**Table S2.** CCD matrix of significant factors and corresponding results for RB5 decolorization by developed consortium.

| **Run order** | **RB5 concentration** | **pH** | **Inoculum size** | **Incubation time** | **Decolorization %** | | **St. Residual** |
| --- | --- | --- | --- | --- | --- | --- | --- |
|  |  |  |  |  | **Experimental** | **Predicted** |  |
| **1** | 0 | 0 | 0 | 0 | 76.98±1.42 | 74.25 | 0.73 |
| **2** | 0 | 0 | 0 | 0 | 71.03±0.32 | 74.25 | - 0.86 |
| **3** | -2 | 0 | 0 | 0 | 85.16±0.84 | 82.32 | 1.09 |
| **4** | -1 | -1 | -1 | 1 | 65.83±1.51 | 68.51 | - 1.03 |
| **5** | -1 | -1 | 1 | 1 | 83.28±0.44 | 83.16 | 0.05 |
| **6** | 0 | 0 | 2 | 0 | 80.54±1.03 | 80.65 | - 0.04 |
| **7** | -1 | 1 | -1 | -1 | 68.00±1.0 | 68.97 | - 0.37 |
| **8** | 0 | 0 | 0 | -2 | 58.34±0.25 | 57.55 | 0.30 |
| **9** | 1 | 1 | 1 | -1 | 59.74±0.73 | 61.57 | - 0.70 |
| **10** | -1 | 1 | 1 | 1 | 93.84±1.29 | 92.54 | 0.50 |
| **11** | -1 | -1 | 1 | -1 | 71.05±2.01 | 72.07 | - 0.39 |
| **12** | 0 | 0 | 0 | 0 | 72.68±1.19 | 74.25 | -0.42 |
| **13** | 1 | -1 | -1 | 1 | 55.73±1.14 | 52.27 | 1.32 |
| **14** | 1 | 1 | 1 | 1 | 85.13±1.63 | 86.28 | - 0.44 |
| **15** | 1 | 1 | -1 | 1 | 66.11±1.95 | 69.60 | - 1.34 |
| **16** | 1 | 1 | -1 | -1 | 49.33±0.33 | 49.10 | 0.09 |
| **17** | 0 | 2 | 0 | 0 | 79.55±0.48 | 74.63 | 1.88 |
| **18** | 0 | 0 | 0 | 0 | 70.93±1.57 | 74.25 | - 0. 89 |
| **19** | -1 | -1 | -1 | -1 | 63.14±2.03 | 61.64 | 0.57 |
| **20** | 0 | 0 | 0 | 0 | 74.38±0.27 | 74.25 | 0.03 |
| **21** | 0 | 0 | 0 | 0 | 76.74±1.06 | 74.21 | 0.67 |
| **22** | 0 | 0 | -2 | 0 | 57.84±0.56 | 53.54 | 1.65 |
| **23** | 0 | 0 | 0 | 0 | 77.02±1.82 | 74.25 | 0.74 |
| **24** | 2 | 0 | 0 | 0 | 50.12±2.27 | 48.74 | 0.51 |
| **25** | 1 | -1 | -1 | -1 | 28.56±0.23 | 34.37 | - 2.23 R |
| **26** | -1 | 1 | -1 | 1 | 73.48±1.21 | 78.44 | - 1.90 |
| **27** | 0 | 0 | 0 | 2 | 92.53±0.78 | 89.13 | 1.30 |
| **28** | 1 | -1 | 1 | 1 | 65.95±1.05 | 69.42 | - 1.36 |
| **29** | -1 | 1 | 1 | -1 | 75.76±1.34 | 78.87 | - 1.19 |
| **30** | 0 | -2 | 0 | 0 | 49.78±0.67 | 50.52 | - 0.28 |
| **31** | 1 | -1 | 1 | -1 | 52.68±0.71 | 47.37 | 2.03 R |

**Table S3.** Cytotoxicity of RB5 dye on epithelial breast cell MCF-12F and skin fibroblast BJ-1.

| **Concentration (%)** | **Cytotoxicity % on breast cell MCF-12F** | | | **Cytotoxicity % on fibroblast BJ-1** | |
| --- | --- | --- | --- | --- | --- |
|  | **RB5** | **Metabolites** | | **RB5** | **Metabolites** |
| 0 | 1.192 ± 0.17 | | 1. 045 ± 0.05 | 1.55 ± 0.21 | 1.57 ± 0.21 |
| 2.5 | 2.61 ± 0.52 | | 1.59 ± 0.23 | 11.13 ± 0.54 | 1.88 ± 0.12 |
| 5 | 5.95 ± 0.26 | | 2.58 ± 0.51 | 14.43 ± 1.39 | 2.53 ± 0.23 |
| 10 | 10.69 ± 1.01 | | 3.78 ± 0.81 | 18.74 ± 0.99 | 3.04 ± 0.15 |
| 25 | 24.35 ± 2.02 | | 3.92 ± 0.79 | 36.72 ± 1.62 | 9.73 ± 1.04 |
| 50 | 55.31 ± 1.65 | | 14.45 ±1.84 | 73.45 ± 1.06 | 16.87 ± 0.91 |
| P value | 0.000 *** | | 0.000 *** | 0.000 *** | 0.000 *** |
| IC50 | 46.30 | | ND | 33.21 | ND |

ND: not detected, Values are mean ± S.D of triplicate measurements of the cytotoxicity rate, *, **, *** significant between treatments at P<0.05, P<0.01, and P<0.001, respectively, using one-way analysis of variance (ANOVA). IC_50_ value = the concentration of the tested solution to produce 50% inhibition of cell growth.


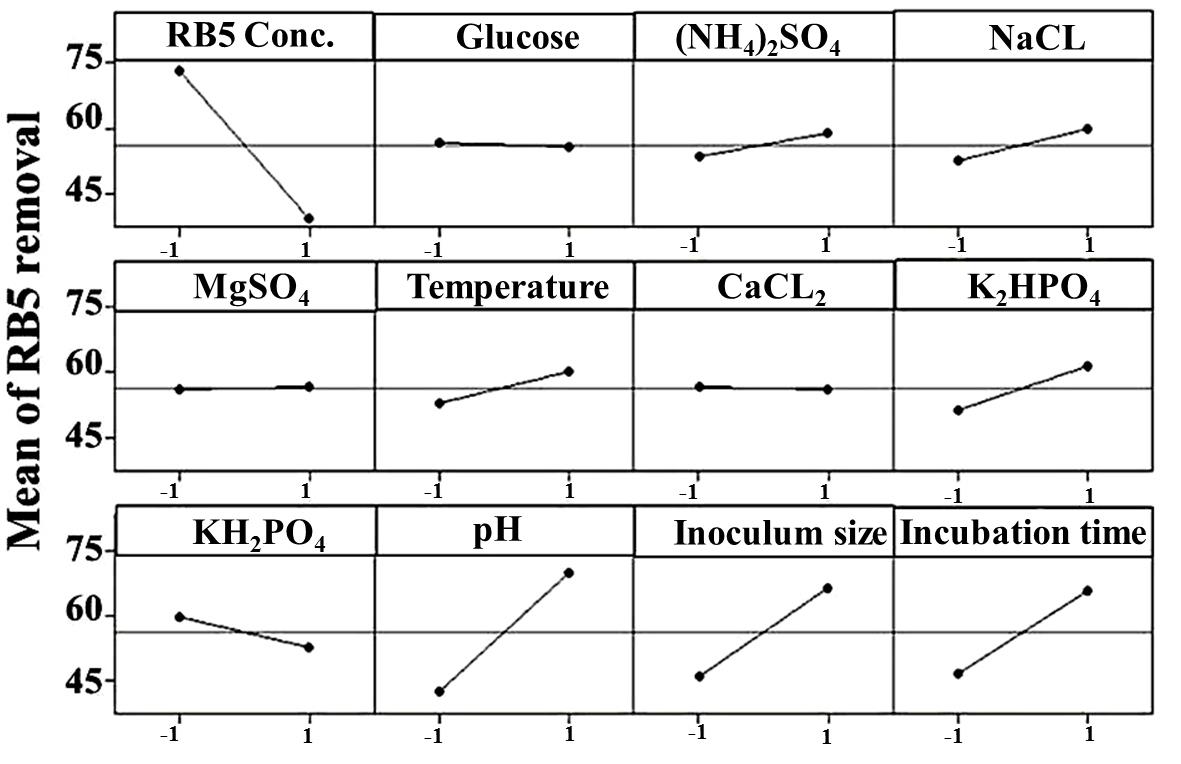


**Fig. S1.** Main effect plot of each variable on RB5 decolorization. When the variable has positive main effect, the response (RB5 removal) increases as the variable is deviated from low to high level and this is clearly noted in case of **(**NH_4_)_2_SO_4_, NaCl, temperature, K_2_HPO_4_, pH**,** inoculum size and incubation time. While, RB5 concentration and KH_2_PO_4_ is changed from high to low level indicating their negative main effect on increasing the response. Other factors such as glucose, MgSO_4_ and CaCl_2_ display very low effect on removal, where the lines are deviated by a small degree from the horizontal baseline.
